# Study variation in estimating Glomerular Filtration Rate (GFR) from serum creatinine, cystatin C, age, and sex

**DOI:** 10.1371/journal.pone.0357209

**Published:** 2026-09-28

**Authors:** Nora Fino, Ben Haaland, Lesley A. Inker, Andrew Levey, Josef Coresh, Ogechi M. Adingwupu, Tom Greene

**Affiliations:** 1 Division of Epidemiology, Department of Internal Medicine, University of Utah Health, Salt Lake City, Utah, United States of America; 2 Pentara Corporation, Millcreek, Utah, United States of America; 3 Department of Medicine, Division of Nephrology, Tufts Medical Center, Boston, Massachusetts, United States of America; 4 Department of Population Health, NYU Langone, New York, New York, United States of America; University of Toronto Department of Laboratory Medicine and Pathobiology, CANADA

## Abstract

**Introduction:**

In routine clinical practice, kidney function is assessed by estimating the glomerular filtration rate (GFR) using equations that combine serum creatinine and/or cystatin C with age and sex. These equations are typically created by pooling datasets from multiple research studies and populations into a single dataset. We investigate implications of study and population variation on the relationship of GFR with its predictor variables.

**Methods:**

We combined data used to develop and validate the 2021 and 2012 The Chronic Kidney Disease Epidemiology Collaboration (CKD-EPI) equations based on creatinine or cystatin C (13,391 individuals in 26 studies). We used random effects modeling to partition the deviations between measured GFR (mGFR) and estimated GFR (eGFR) into four components: predictable variation between studies of CKD vs. non-CKD populations, non-predictable variation between studies, patient-level error in eGFR within studies, and measurement error in mGFR.

**Results:**

Overall, the average eGFR was similar across different levels of the predictor variables between the random effect models and the original pooled CKD-EPI equations. However, the random effect model demonstrated substantial variation between studies, both between and within CKD and non-CKD populations. After accounting for the population type (CKD or non-CKD), approximately 10–40% of the deviations between eGFR and mGFR was attributable to study variation, 20–40% to measurement error in mGFR, and 20–70% to patient level errors in eGFR. Both pooled and random‐effects models generalized well across studies, whereas using equations derived from a single study leads to substantial overfitting and larger prediction errors.

**Conclusions:**

Single‐study GFR equations may underestimate cross-study prediction error by ignoring study‐level variability, especially in non-CKD populations. Our results largely corroborate the use of pooled datasets and the current form of the CKD-EPI equations. Despite measureable study-level heterogeneity, the pooled CKI-EPI apparach remains a pragmatic and broadly available solution for GFR estimation.

## Introduction

The glomerular filtration rate (GFR) is widely considered the best indicator of kidney function but it cannot be measured directly. Instead, standard clinical practice is to calculate an estimated glomerular filtration rate (eGFR) using estimating equations based on serum concentrations of endogenous filtration markers, usually creatinine and/or cystatin C, and demographic variables such as age and sex. Current guidelines recommend creatinine-based eGFR (eGFRcr) for general use, with cystatin C–based eGFR (eGFRcys) or combined eGFRcr-cys used when greater accuracy is needed [1]. The Chronic Kidney Disease Epidemiology Collaboration (CKD-EPI) equations for estimating GFR [2] are the most common equations used for adults in practice [1,3].

The CKD-EPI equations are effective, with 80–90% of patients having eGFRs within 30% of their mGFR in both the general population and patients with CKD [2]. These equations were developed using pooled data from diverse groups, including patients with chronic kidney disease or diabetes, healthy kidney donor candidates, and individuals from North America and Europe. Combining across multiple datasets ensure that broad distributions of creatinine, cystatin C, age, and sex were represented. Pooling data across studies necessarily averages over differences between source studies, such as how GFR was measured or which patients were included. These factors may introduce variability in the marker-mGFR relationship that is not explicitly represented in the final clinical equation. Thus, even when pooled equations perform well for broad clinical use, it remains useful to quantify the extent of study-level variation and its implications for prediction uncertainty.

Additionally, the relationship between GFR and its predictors may depend on clinical context. Just as predictive values of diagnostic tests often differ between screening populations and patients with known disease, the relationship between measured GFR with its predictor variables may vary across settings. For example, the relationship between mGFR and creatinine or cystatin C differs between studies focusing on healthy individuals, kidney donor candidates or other populations having few patients with substantially reduced GFR compared to studies whose entry criteria restrict to patients with CKD, as shown by Murata et al [4].

Our overall goal in this paper is to investigate the implications of study variation in the relationship of GFR with the predictor variables serum creatinine or cystatin C, age, and sex. We apply random effects models that account for study-to-study variation to quantify the magnitude and consquences of this variation and to assess whether it materially challenges the pooled approach.

## Methods

### Study populations & clinical context

We used data from research studies and clinical populations (S1 File Study Collaborators and S2 File: Table S1) associated with the CKD-EPI database. Specifically, we included data used to develop and validate the currently used CKD-EPI eGFR equations [2]: CKD-EPI 2009 and CKD-EPI 2021 creatinine equations (10 studies, 8,254 participants), CKD-EPI 2012 cystatin C equation (13 studies, 5,352 participants), and CKD-EPI 2021 creatinine and cystatin C equations (12 studies, 4,050 participants). In total, there were 13,398 unique individuals across 27 studies, as several studies overlap between the 2009, 2012, and 2021 datasets. We excluded one study (7 participants) due to its small sample size; therefore, our final analytic dataset contained 13,391 individuals in 26 studies. All studies received approval from the institutional review boards of the participating institutions.

All studies have mGFR determined by urinary or plasma clearance of exogenous filtration markers; we calibrated mGFR using plasma clearance of iohexol by increasing it by 5% to align with other methods [2,5,6]. Creatinine and cystatin C measurements were standardized across studies (Supplemental Methods). To summarize the clinical contexts of the studies, we categorized the studies into two distinct groups: studies that explicitly enrolled individuals with CKD (CKD populations) and studies of individuals enrolled for other reasons (non-CKD populations). The non-CKD classification should not be interpreted as indicating that all participants were free of CKD; rather, these studies were not designed to enroll participants on the basis of established CKD status, and some individuals within these populations may have met clinical criteria for CKD.

### Statistical modeling framework

Our primary goal is to characterize how the relationship between mGFR and its predictor variables varies across studies and to evaluate the impact of this variation on GFR estimation. The CKD-EPI equations estimated log-transformed mGFR from log-transformed creatinine and/or cystatin C, age, and sex, including two linear splines for creatinine and/ or cystatin C [2]. The general structure of these equations is provided in the Supplemental Methods. For simplicity, we first focus on study variation in the relationship of mGFR with creatinine, age, and sex; analyses for cystatin C with demographic variables follow similarly.

Next, we applied a random effects model for eGFRcr. Random effects modeling represents a compromise between the complete pooling (as in the original equation) approach and the no-pooling approach (as in the study-specific models) [7]. Using a random effects model, we can explicitly distinguish the variation in mGFR between and within the different studies. Because we hypothesized that the mGFR–creatinine relationship differs between CKD and non-CKD populations, we allowed the creatinine coefficients to vary by study type. More details are provided in the Supplement, but the general form of the random effects models can be decomposed into five parts:


$$\begin{array}{cc} \text{log}(mGFR) & =\underset{\left(\text{1}\right)}{\underbrace{\left[\text{population equation}\right]}}+\underset{\left(\text{2}\right)}{\underbrace{\left[\text{deviation in the population equation for non}-\text{CKD studies vs}.\text{ CKD studies}\right]}}\\ & +\underset{\left(\text{3}\right)}{\underbrace{\left[\text{unpredictable study deviations}\right]}}+\underset{\left(\text{4}\right)}{\underbrace{\left[\text{error in predicting true GFR within a given study}\right]}}\\ & +\underset{\left(\text{5}\right)}{\underbrace{\left[\text{error as a result of measurement error in mGFR}\right]}} \end{array}$$

Where term (1) estimates the mean log-mGFR as a function of creatinine, sex, and age across all studies, Term (2) applies a fixed adjustment to allow for deviations between studies of CKD vs. predominantly non-CKD populations, term (3) comprises the random intercepts and slopes for log-creatinine, age, and sex, capturing additional study-specific variation around the adjusted equations in Terms (1) and (2) Term (4) reflects the within-study prediction error (with its standard deviation allowed to differ between CKD and non-CKD studies), and Term (5) reflects assay-related noise in mGFR itself.

The random-effects structure was selected to capture study-specific deviations in the overall level of mGFR and in the associations of mGFR with the main filtration marker, age, and sex, while maintaining a parsimonious model that could be estimated across studies of varying sample size. We included random intercepts and random slopes for log creatinine or log cystatin C, age, and sex. We did not include separate random effects for each spline term because, within many studies, creatinine or cystatin C values were concentrated predominantly on one side of the knot; estimating separate study-specific random effects for each spline segment would therefore be unstable. Instead, a single random slope for the filtration marker was used to represent study-specific tilting of the marker–mGFR relationship

### Variance decomposition & model evaluation

Using the final random effects models, we partitioned the total residual variance in log mGFR that remained after accounting for the fixed effects terms into three parts: between-study variation, within-study variation, and measurement error in mGFR (see Supplemental Methods). We assumed that log mGFR provides an unbiased estimate of log true GFR, and that the measurement error variance in log mGFR is 0.015 based on previous studies of repeated GFR measurements in the African American Study of Kidney Disease and Hypertension [8] (AASK) and The Modification of Diet in Renal Disease [9] (MDRD) studies (Table S2 in S2 File). Given the log scale, this measurement error variance corresponds to a coefficient of variation (CV) of about 12.3% for the untransformed mGFR (i.e., on average, the measurement varies by about 12% relative to the true value; calculated as $\sqrt{\text{exp}(\text{0}.\text{0}\text{1}\text{5})-\text{1}}=\text{1}\text{2}.\text{3}\%$). The variance decompositions should be interpreted as model-based estimates of the relative contributions of between-study variation, within-study patient-level variation, and mGFR measurement error; as such, summaries focus on the relative magnitude of these sources of variation under the fitted model.

Lastly, we empirically assessed the impact of study variation on the performance of GFR estimating equations derived from single studies versus those pooled across multiple studies. Error was summarized using the root mean square error (RMSE) for predicted vs. measured GFR. For each study, we first computed RMSE of study-specific models using patient-level cross-validation within that study. We then assessed cross-study generalizability using a leave-one-study-out internal validation approach, fitting the model on all other studies combined and applying it to the held-out study.

Next, we evaluated a pooled estimate that explicitly accounted for study effects by fitting the random-effects model (defined as above) to all other studies combined and applying the fixed-effect coefficients to the target study. To examine the performance of study-specific equations, we calculated the average RMSE obtained when applying each study-specific equation to all remaining studies, and repeated this analysis within study classifications (CKD vs. non-CKD populations). Finally, we assessed overfitting by calculating two ratios: the ratio of the average RMSE of single-study equations to the average within-study cross-validation RMSE, and the ratio of the average RMSE of single-study equations applied within the same study classification to the corresponding within-study cross-validation RMSE.

## Results

### Descriptive analysis

Across all studies, all individuals (13,391) have mGFR and creatinine, but only a subset of 7,496 individuals have cystatin C available. The 26 studies are summarized in Tables S3 and S4 in S2 File, and Fig 1 provides the distribution of mGFR, creatinine, and cystatin C by study. Thirteen of these studies, comprising 5,268 individuals, represented non-CKD populations. These studies tended to have high mGFR with low creatinine and cystatin C, with an overall mean mGFR of 96.6 ml/min/1.73m^2^ (standard deviation (SD) 27.0), mean creatinine of (0.9 [SD 0.3] mg/dL), and mean cystatin C (0.9 [SD 0.3] mg/L). The remaining 13 studies (comprising 8,123 individuals) were of CKD populations. Individuals from these studies tended to have a lower mean mGFR (50.2 [SD 27.7]ml/min/1.73m^2^), higher mean creatinine (1.9 [SD 1.2] mg/dL), and higher mean cystatin C (1.5 [SD 0.8] mg/L) compared to the studies of non-CKD populations. Study-specific regression lines for eGFR based on creatinine or cystatin C are shown in Fig 2, and coefficients for the study-specific regressions can be found in Table S5 in S2 File. We observed considerable variation in the coefficients by study.

**Fig 1:**
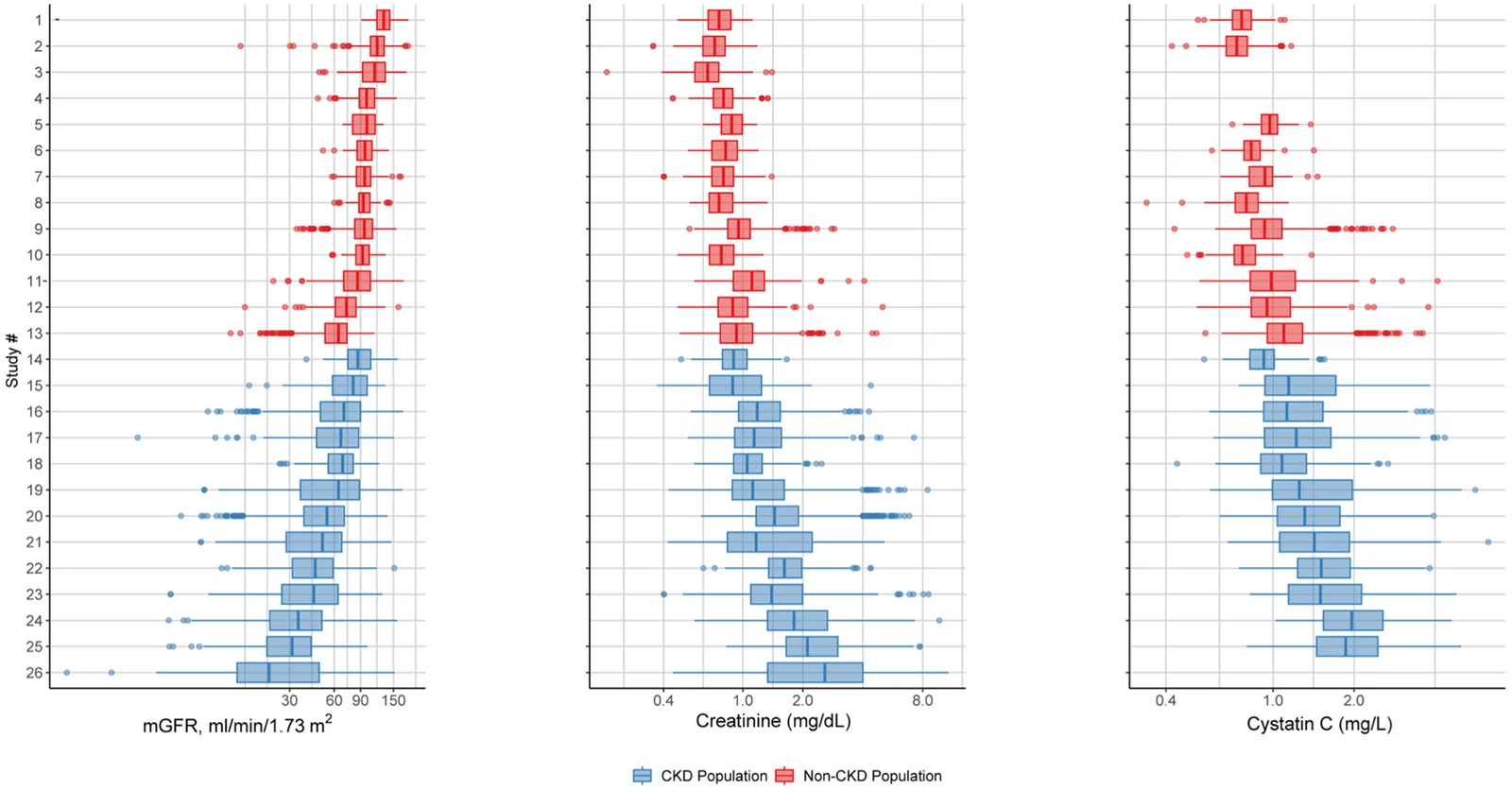
Distribution of mGFR, creatinine, and cystatin C by study. Colors denote studies of non-CKD populations vs. CKD populations. Studies are sorted by mean mGFR (ml/min/1.73m2). Cystatin C was not reported in studies 3,4, and 26. Abbreviations: mGFR: measured Glomerular Filtration Rate.

**Fig 2:**
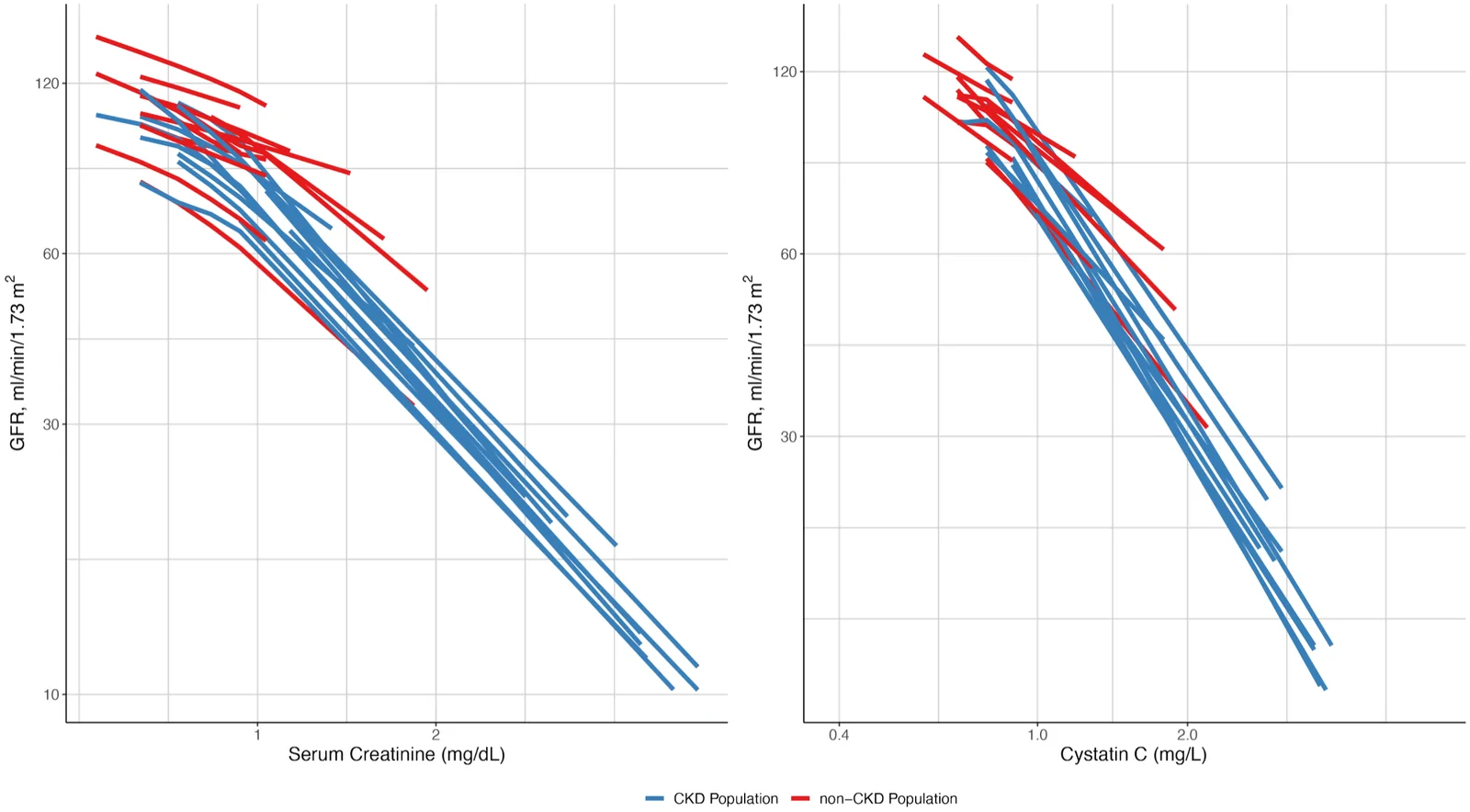
Study-specific models for each study. Each line represents a regression line fit in each study separately. Colors denote studies of non-CKD populations vs. CKD populations. Data is shown for creatinine/cystatin C values between the 2.5th and 97.5th percentile for each study. All estimates are averages across participants, with their corresponding age and sex, in a given study.

### Between study analyses

The estimated coefficients of the predictor variables within each study were largely similar when estimated under the random effects model via best linear unbiased prediction (BLUP) as when estimated in separate analyses of each study (Table 1). BLUPs are study-specific coefficient estimates from the random-effects model that combine information from the individual study with information from the full pooled dataset, thereby stabilizing estimates for smaller studies while preserving study-level deviations. Coefficients, as well as estimates of the random effect covariances matrix, for each model term in the random effect models are provided in Tables S6 and S7 in S2 File.

**Table 1:** Population mean and predicted study-specific coefficients from the fitted random effects model using creatinine (model n = 13,391, Panel A) or cystatin C (model n = 7,496, Panel B). Studies 1 through 13 denote studies of non-CKD populations and studies 14 through 26 studies of CKD populations. Lower slopes refer to slopes prior to a given knot point, while higher slopes refer to slopes after the knot point. Cells marked with “–” indicate that the coefficient could not be estimated for that study. Studies 3, 4, and 26 did not report cystatin C measurements.

**A**						
**Study #**	**Study Sample Size**	**Coefficients for Creatinine (95% CI)**			**Coefficient for Age,** **per 1 year (95% CI)**	**Coefficient for Female Sex (95% CI)**
		**Slope for lower creatinine in males**	**Slope for lower creatinine in females**	**Slope for higher creatinine**		
**2021 CKD-EPI Creatinine**		**−0.302 (−0.403, −0.202)**	**−0.241 (−0.344,** **−0.138)**	**−1.200 (−1.211,** **−1.189)**	**0.9938 (0.9935, 0.9942)**	**1.012 (1.000, 1.023)**
1	Moderate (200–499)	−0.328 (−0.448, −0.208)	−0.338 (−0.460, −0.216)	−0.568 (−0.685, −0.452)	0.996 (0.995,0.998)	0.974 (0.931,1.020)
2	Very large (≥ 1,000)	−0.242 (−0.318, −0.167)	−0.252 (−0.332, −0.171)	−0.482 (−0.564, −0.400)	0.996 (0.995,0.998)	1.047 (1.017,1.078)
3	Small (50–199)	−0.467 (−0.571, −0.364)	−0.477 (−0.573, −0.380)	−0.707 (−0.813, −0.602)	0.994 (0.992,0.995)	0.917 (0.872,0.963)
4	Moderate (200–499)	−0.247 (−0.357, −0.137)	−0.257 (−0.368, −0.145)	−0.487 (−0.580, −0.394)	0.995 (0.994,0.996)	1.081 (1.041,1.123)
5	Very small (< 50)	−0.379 (−0.592, −0.166)	−0.389 (−0.604, −0.173)	−0.619 (−0.823, −0.415)	0.993 (0.991,0.996)	0.977 (0.901,1.059)
6	Small (50–199)	−0.349 (−0.523, −0.175)	−0.359 (−0.534, −0.183)	−0.589 (−0.754, −0.424)	0.993 (0.992,0.995)	0.990 (0.928,1.056)
7	Large (500–999)	−0.175 (−0.277, −0.074)	−0.185 (−0.282, −0.088)	−0.415 (−0.499, −0.332)	0.995 (0.994,0.996)	1.071 (1.035,1.108)
8	Small (50–199)	−0.253 (−0.401, −0.106)	−0.263 (−0.415, −0.111)	−0.493 (−0.633, −0.353)	0.994 (0.993,0.996)	1.015 (0.959,1.073)
9	Large (500–999)	−0.496 (−0.579, −0.413)	−0.506 (−0.606, −0.406)	−0.736 (−0.792, −0.680)	0.993 (0.992,0.994)	0.958 (0.880,1.044)
10	Moderate (200–499)	−0.298 (−0.428, −0.168)	−0.308 (−0.436, −0.179)	−0.538 (−0.653, −0.422)	0.994 (0.992,0.995)	1.013 (0.968,1.060)
11	Moderate (200–499)	−0.600 (−0.710, −0.490)	−0.610 (−0.727, −0.493)	−0.840 (−0.914, −0.766)	0.991 (0.990,0.993)	0.932 (0.889,0.977)
12	Moderate (200–499)	−0.572 (−0.673, −0.471)	−0.582 (−0.688, −0.475)	−0.812 (−0.884, −0.740)	0.989 (0.988,0.991)	0.852 (0.818,0.887)
13	Large (500–999)	−0.693 (−0.783, −0.603)	−0.703 (−0.798, −0.607)	−0.933 (−0.973, −0.893)	0.988 (0.986,0.989)	0.890 (0.865,0.917)
14	Small (50–199)	0.035 (−0.187, 0.257)	0.039 (−0.228, 0.307)	−0.960 (−1.098, −0.822)	0.996 (0.994,0.998)	1.066 (1.000,1.137)
15	Very small (< 50)	−0.143 (−0.372, 0.085)	−0.139 (−0.397, 0.119)	−1.138 (−1.285, −0.992)	0.994 (0.991,0.996)	1.004 (0.914,1.103)
16	Large (500–999)	−0.263 (−0.449, −0.076)	−0.258 (−0.510, −0.006)	−1.258 (−1.309, −1.206)	0.994 (0.993,0.996)	1.064 (1.024,1.104)
17	Moderate (200–499)	−0.176 (−0.364, 0.012)	−0.172 (−0.427, 0.083)	−1.171 (−1.238, −1.105)	0.994 (0.992,0.996)	1.009 (0.956,1.066)
18	Moderate (200–499)	0.030 (−0.166, 0.225)	0.034 (−0.226, 0.295)	−0.965 (−1.050, −0.880)	0.994 (0.993,0.996)	1.017 (0.973,1.064)
19	Moderate (200–499)	−0.195 (−0.380, −0.010)	−0.190 (−0.440, 0.059)	−1.190 (−1.239, −1.141)	0.994 (0.993,0.995)	1.030 (0.982,1.081)
20	Very large (≥ 1,000)	−0.082 (−0.270, 0.106)	−0.078 (−0.330, 0.175)	−1.077 (−1.106, −1.048)	0.995 (0.994,0.996)	0.984 (0.958,1.011)
21	Small (50–199)	−0.110 (−0.299, 0.079)	−0.106 (−0.365, 0.153)	−1.105 (−1.184, −1.027)	0.993 (0.990,0.994)	0.977 (0.909,1.049)
22	Large (500–999)	−0.215 (−0.409, −0.020)	−0.210 (−0.467, 0.047)	−1.210 (−1.268, −1.151)	0.995 (0.994,0.996)	1.013 (0.976,1.053)
23	Large (500–999)	−0.136 (−0.321, 0.049)	−0.131 (−0.379, 0.116)	−1.131 (−1.173, −1.089)	0.992 (0.991,0.994)	0.995 (0.955,1.038)
24	Very large (≥ 1,000)	−0.163 (−0.351, 0.025)	−0.159 (−0.410, 0.092)	−1.158 (−1.184, −1.133)	0.995 (0.994,0.995)	0.991 (0.964,1.019)
25	Moderate (200–499)	−0.127 (−0.323, 0.069)	−0.122 (−0.380, 0.135)	−1.122 (−1.183, −1.060)	0.994 (0.993,0.996)	0.953 (0.905,1.004)
26	Very large (≥ 1,000)	−0.106 (−0.293, 0.080)	−0.102 (−0.352, 0.148)	−1.101 (−1.125, −1.078)	0.994 (0.993,0.994)	1.031 (0.999,1.064)
**Mean, Studies 1–13**	5,757	−0.391 (−0.504, −0.278)	−0.379 (−0.495, −0.262)	−0.633 (−0.719, −0.547)	0.994 (0.993,0.994)	0.992 (0.972, 1.012)
**Mean, Studies 14–26**	8,123	−0.144 (−0.334, 0.046)	−0.140 (−0.391, 0.112)	−1.139 (−1.195, −1.083)		
Between-Study SD of coefficient		0.136			0.062	0.002
**B**						
**Study #**	**Study Sample Size**	**Coefficients for Cystatin C (95% CI)**		**Coefficient for Age,** **per 1 year (95% CI)**	**Coefficient for Female Sex (95% CI)**	
		**Slope for lower values**	**Slope for higher values**			
**2021 CKD-EPI Cystatin C**		**−0.499 (−0.610, −0.388)**	**−1.328 (−1.344,** **−1.312)**	**0.9962 (0.9957, 0.9966)**	**0.932 (0.921, 0.944)**	
1	Moderate (200–499)	−0.471 (−0.622, −0.321)	−0.808 (−0.963, −0.654)	0.996 (0.994,0.998)	0.903 (0.872,0.935)	
2	Very large (≥ 1,000)	−0.362 (−0.443, −0.280)	−0.699 (−0.809, −0.589)	0.996 (0.995,0.998)	0.959 (0.941,0.977)	
3	Small (50–199)	–	–	–	–	
4	Moderate (200–499)	–	–	–	–	
5	Very small (< 50)	−0.298 (−0.554, −0.041)	−0.635 (−0.877, −0.392)	0.994 (0.991,0.996)	0.937 (0.879,0.998)	
6	Small (50–199)	−0.470 (−0.688, −0.253)	−0.807 (−1.012, −0.603)	0.996 (0.994,0.998)	0.928 (0.884,0.974)	
7	Large (500–999)	−0.055 (−0.399, 0.288)	−1.133 (−1.352, −0.914)	0.995 (0.993,0.998)	0.944 (0.891,1.000)	
8	Small (50–199)	−0.265 (−0.415, −0.115)	−0.602 (−0.756, −0.448)	0.995 (0.994,0.997)	0.942 (0.904,0.982)	
9	Large (500–999)	−0.352 (−0.468, −0.235)	−0.689 (−0.738, −0.639)	0.995 (0.993,0.996)	0.938 (0.869,1.013)	
10	Moderate (200–499)	−0.476 (−0.610, −0.343)	−0.813 (−0.953, −0.674)	0.996 (0.995,0.998)	0.945 (0.914,0.977)	
11	Moderate (200–499)	−0.520 (−0.648, −0.392)	−0.857 (−0.932, −0.782)	0.996 (0.994,0.998)	0.937 (0.900,0.976)	
12	Moderate (200–499)	−0.491 (−0.616, −0.367)	−0.828 (−0.899, −0.758)	0.996 (0.994,0.998)	0.876 (0.849,0.904)	
13	Large (500–999)	−0.666 (−0.789, −0.543)	−1.003 (−1.044, −0.962)	0.996 (0.994,0.998)	0.934 (0.916,0.953)	
14	Small (50–199)	0.170 (−0.112, 0.453)	−0.907 (−1.065, −0.750)	0.994 (0.991,0.996)	0.947 (0.902,0.995)	
15	Very small (< 50)	−0.080 (−0.403, 0.242)	−1.158 (−1.336, −0.980)	0.995 (0.992,0.998)	0.950 (0.886,1.018)	
16	Large (500–999)	−0.234 (−0.505, 0.036)	−1.312 (−1.376, −1.248)	0.998 (0.995,1.000)	0.974 (0.934,1.016)	
17	Moderate (200–499)	−0.168 (−0.441, 0.104)	−1.246 (−1.312, −1.180)	0.996 (0.994,0.998)	0.927 (0.886,0.971)	
18	Moderate (200–499)	0.165 (−0.101, 0.431)	−0.913 (−0.984, −0.842)	0.996 (0.995,0.998)	0.905 (0.873,0.938)	
19	Moderate (200–499)	−0.262 (−0.535, 0.010)	−1.340 (−1.395, −1.285)	0.998 (0.997,1.000)	0.949 (0.912,0.988)	
20	Very large (≥ 1,000)	−0.068 (−0.341, 0.205)	−1.146 (−1.186, −1.106)	0.997 (0.996,0.999)	0.924 (0.898,0.951)	
21	Small (50–199)	−0.302 (−0.590, −0.014)	−1.380 (−1.479, −1.281)	0.997 (0.994,0.999)	0.914 (0.862,0.970)	
22	Large (500–999)	−0.189 (−0.470, 0.092)	−1.267 (−1.340, −1.193)	0.998 (0.997,1.000)	0.924 (0.887,0.963)	
23	Large (500–999)	−0.751 (−0.912, −0.590)	−1.088 (−1.234, −0.942)	0.998 (0.996,1.000)	0.974 (0.930,1.021)	
24	Very large (≥ 1,000)	−0.216 (−0.499, 0.067)	−1.294 (−1.368, −1.220)	1.000 (0.998,1.002)	0.907 (0.868,0.946)	
25	Moderate (200–499)	−0.205 (−0.486, 0.076)	−1.283 (−1.347, −1.218)	0.999 (0.998,1.001)	0.849 (0.812,0.887)	
26	Very large (≥ 1,000)	–	–	–	–	
**Mean, Studies 1–2, 5–13**	5,268	−0.480 (−0.609, −0.353)	−0.817 (−0.923, −0.712)	0.996 (0.995,0.997)	0.930 (0.912, 0.949)	
**Mean, Studies 14–25**	3,639	−0.109 (−0.388, 0.169)	−1.187 (−1.273, −1.101)			
Between-Study SD of coefficient		0.162		0.002	0.039	

Abbreviations: SD: standard deviation.

In the creatinine-based model, when comparing estimates across studies, we observe variation in the log creatinine coefficients not only between the two classes of studies but also considerable variation within each class (Fig 3). For example, the mean slope for log creatinine after the knot was −0.633 (95% CI −0.719, −0.547) in non-CKD studies and −1.139 (95% CI −1.195, −1.083) in CKD studies. Within CKD studies, the predicted slope for higher creatinine values ranged from −0.960 (95% CI −1.098, −0.822) to −1.258 (95% CI −1.309, −1.206). In fact, with a between‐study SD of 0.136, 95% of study‐specific slopes lie within ±1.96 × 0.136 (±0.267) of their population means. We also observed considerable variation by sex, with 95% of sex coefficient deviations between −0.122 and 0.122 on the log scale. The results in the cystatin C-based model were similar, if not more pronounced: we found that 95% of study-specific cystatin C deviations fell between −0.318 and 0.318. Variation by sex was similar to the creatinine-based model.

**Fig 3:**
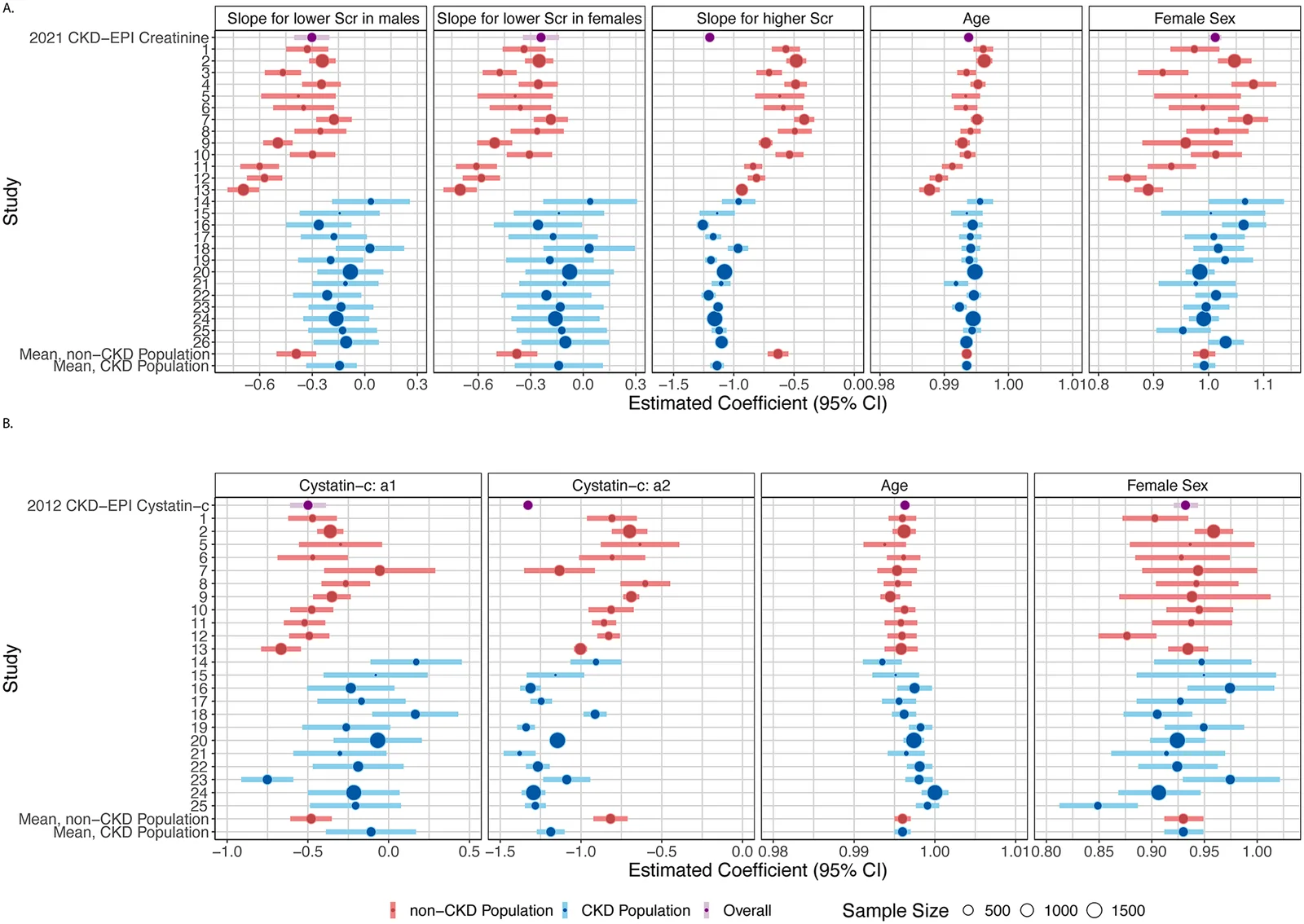
Forest plot of CKD-EPI equation coefficients, population averaged coefficients from the random effects model, and predicted study-specific coefficients from the random effects models for creatinine (Panel A) and cystatin C (Panel B). Data represents the point estimate and 95% confidence interval. Colors denote studies of non-CKD populations vs. CKD populations. Studies are sorted by mean mGFR (ml/min/1.73m^2^). For study-specific estimates, the size of the point represents the sample size of the study. Lower slopes refer to slopes prior to a given knot point, while higher slopes refer to slopes after the knot point. Studies 3, 4, and 26 did not report cystatin C measurements.

### Variation in measured GFR (log scale)

For individuals aged 50 years in studies of non-CKD populations, the estimated total residual variance ranged from 0.032 to 0.038 across creatinine levels and both sexes (Fig 4). Approximately 40–45% of this variation was due to measurement error in mGFR, 25–30% to within-study variation (error from using predictor variables within a single study), and the remaining 25–35% to between-study differences. By contrast, in CKD populations, total residual variance was roughly double, ranging from 0.066 to 0.081. Most of this increase (55–65%) was attributable to within-study variation, with 10–30% explained by between-study differences and about 20% by measurement error. Results were generally similar for individuals ages 25 and 75 (data not shown) and the cystatin C–based model (Fig 4).

**Fig 4:**
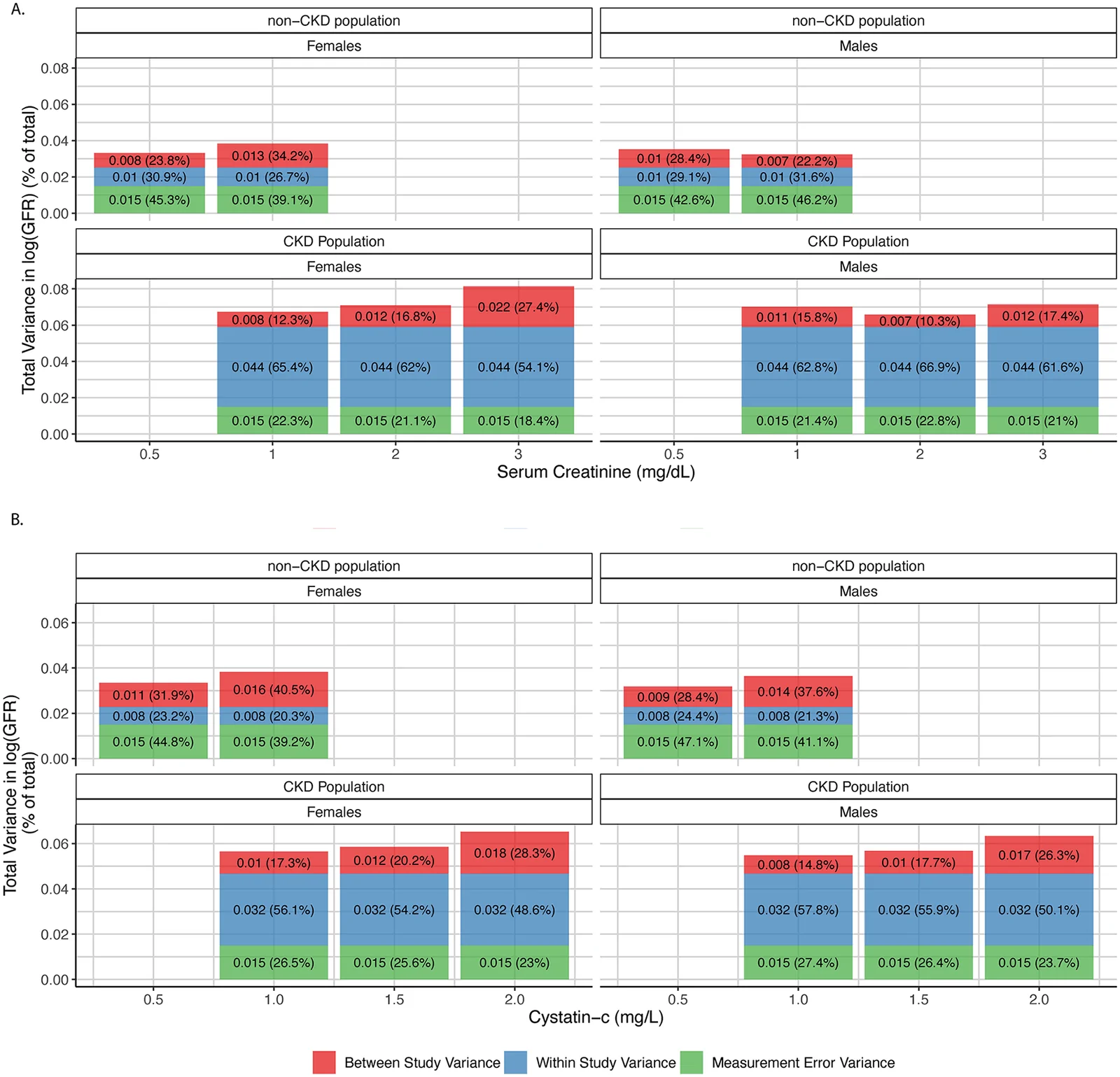
Estimates of the sources of variation in log mGFR. Using either the random effects model based on creatinine (Panel A) or cystatin C (Panel B), we partitioned the total variance into three components: between study variation, within-study variation, and measurement error in mGFR. The heights of the bars represent the total variance in log eGFR, and within each bar, we provided the variance for each component and the percentage of the total variance it represents.

A. Estimates are provided for individuals aged 50 for each sex and various creatinine levels. Variance estimates are provided for a hypothetical study with 50% females, a mean age of 50, and a mean creatinine of 0.8 mg/dL for a non-CKD study and 1.8 mg/dL for a CKD study.
B. Estimates are provided for individuals aged 50 for each sex and various cystatin C levels. Variance estimates are provided for a hypothetical study with 50% females, a mean age of 50, and a mean cystatin C of 0.8 mg/L for a non-CKD and 1.4 mg/L for a CKD study.

We observed broad agreement between the pooled cystatin C equations and the population-averaged regression equations from the random-effects models (Fig 5). In the creatinine model, the pooled equation has a slightly shallower slope before the knot, particularly in females, and a slightly steeper slope after the knot, but overall estimates were very similar. The pooled cystatin C equation likewise showed a slightly steeper slope after the knot compared to our random-effects model. Notably, there is overlap between the two study classes for creatinine values between roughly 0.7 and 1.2 mg/dL. For females in this range near the knot points, the slope of creatinine was −0.633 (95% CI −0.719, −0.547) in non-CKD populations and −1.139 (95% CI −1.195, −1.083) in CKD populations. At a creatinine of 1 mg/dL, this translated to a roughly 25% difference in estimated mean mGFR (61.8 vs. 77.7 ml/min/1.73m^2^ for CKD vs. non-CKD populations, respectively). For cystatin C, the difference was smaller but still notable: at cystatin C of 1 mg/L, estimated mean mGFR was 72.4 ml/min/1.73m^2^ in CKD studies vs. 78.8 ml/min/1.73m^2^ in non-CKD studies. The wide confidence and prediction intervals underscore the uncertainty in predicting GFR for a new individual. For example, for a female with creatinine of 2.0 mg/dL, the estimated mean mGFR was 28.8 ml/min/1.73m^2^, with a prediction interval of 17.7–44.6 ml/min/1.73m^2^ for true GFR and 16.7–47.3 ml/min/1.73m^2^ for mGFR, representing 2.5- and 2.8-fold ranges, respectively. Similarly, for a female with cystatin C of 2.0 mg/L, the estimated mean mGFR was 31.8 ml/min/1.73m^2^, with prediction intervals of 20.5–49.3 (true GFR) and 19.3–52.4 (mGFR).

**Fig 5:**
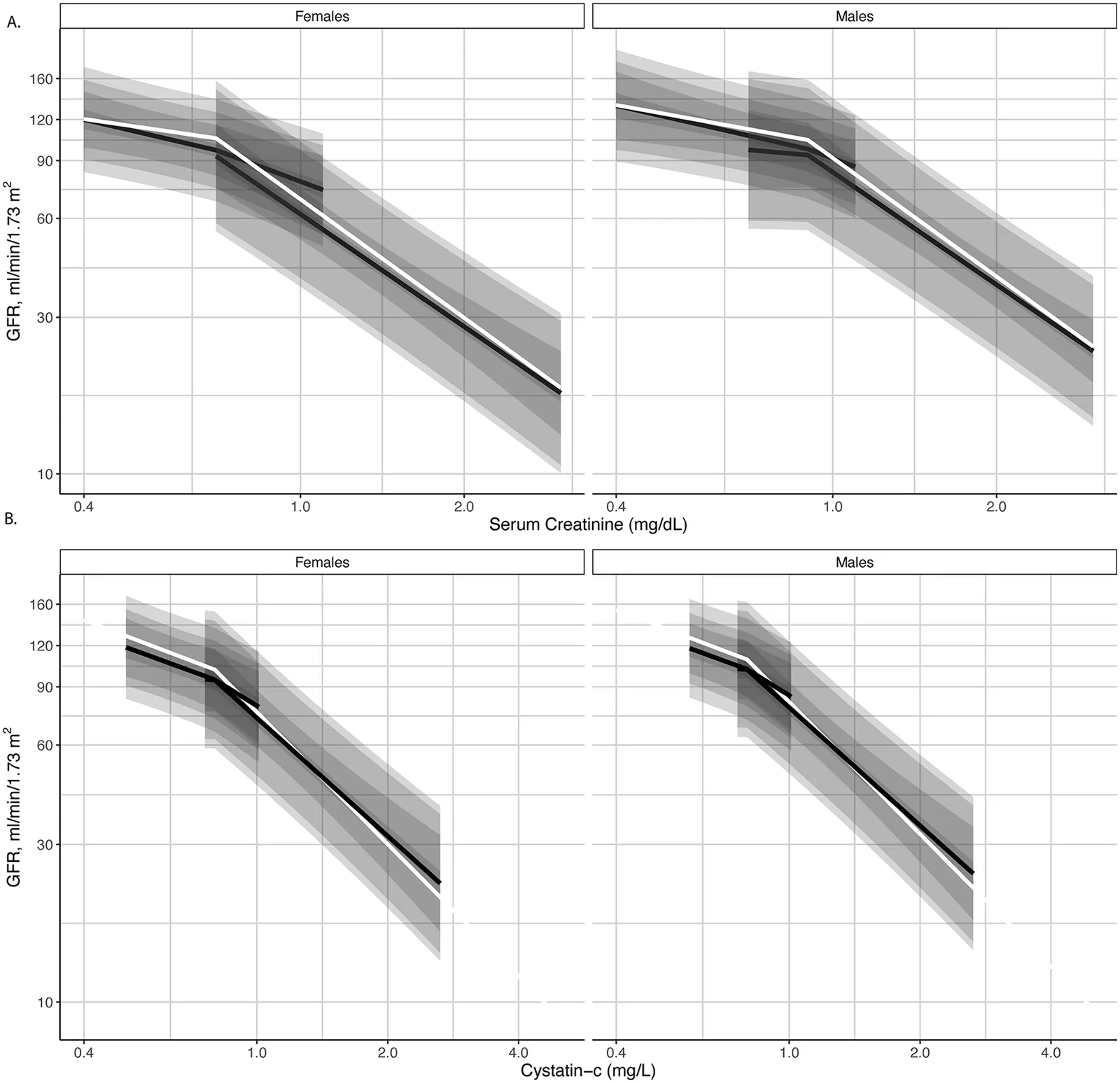
Estimated GFR by creatinine (Panel A) or cystatin C (Panel B) for males and females for individuals aged 50. The black lines represent the mean regression line from the corresponding mixed model for studies of non-CKD populations and CKD populations, while the white line represents the corresponding CKD-EPI equation. The shaded bands, starting from the innermost and moving outward, represent the confidence interval for the mean mGFR across the population of studies given the fixed effects; the prediction intervals for study-specific mean mGFR, given the fixed effects. The prediction interval for true GFR at the patient level, given the fixed effects and accounting for both study and individual-level variation; and the prediction intervals for mGFR at the patient level, given the fixed effects and accounting for both study and individual-level variation.

Across the 26 studies, within‐study cross–validation RMSEs ranged from 0.11 to 0.34 (Table S8 in S2 File) for creatinine based models, with CKD studies tending toward higher RMSEs. When a pooled model (trained on all other studies) was applied to each held‐out study, RMSEs increased modestly, typically by 0.02 to 0.08, relative to the within‐study RMSE. Incorporating study effects via a random‐effects model yielded very similar RMSEs to the pooled fixed‐effect approach. In contrast, applying single‐study equations to a new study consistently produced larger errors (Fig 6). We found that the estimates lay well above the diagonal, sometimes by a factor of two or more, indicating that a model developed and applied within the same small study will underestimate its cross-study prediction error.

**Fig 6:**
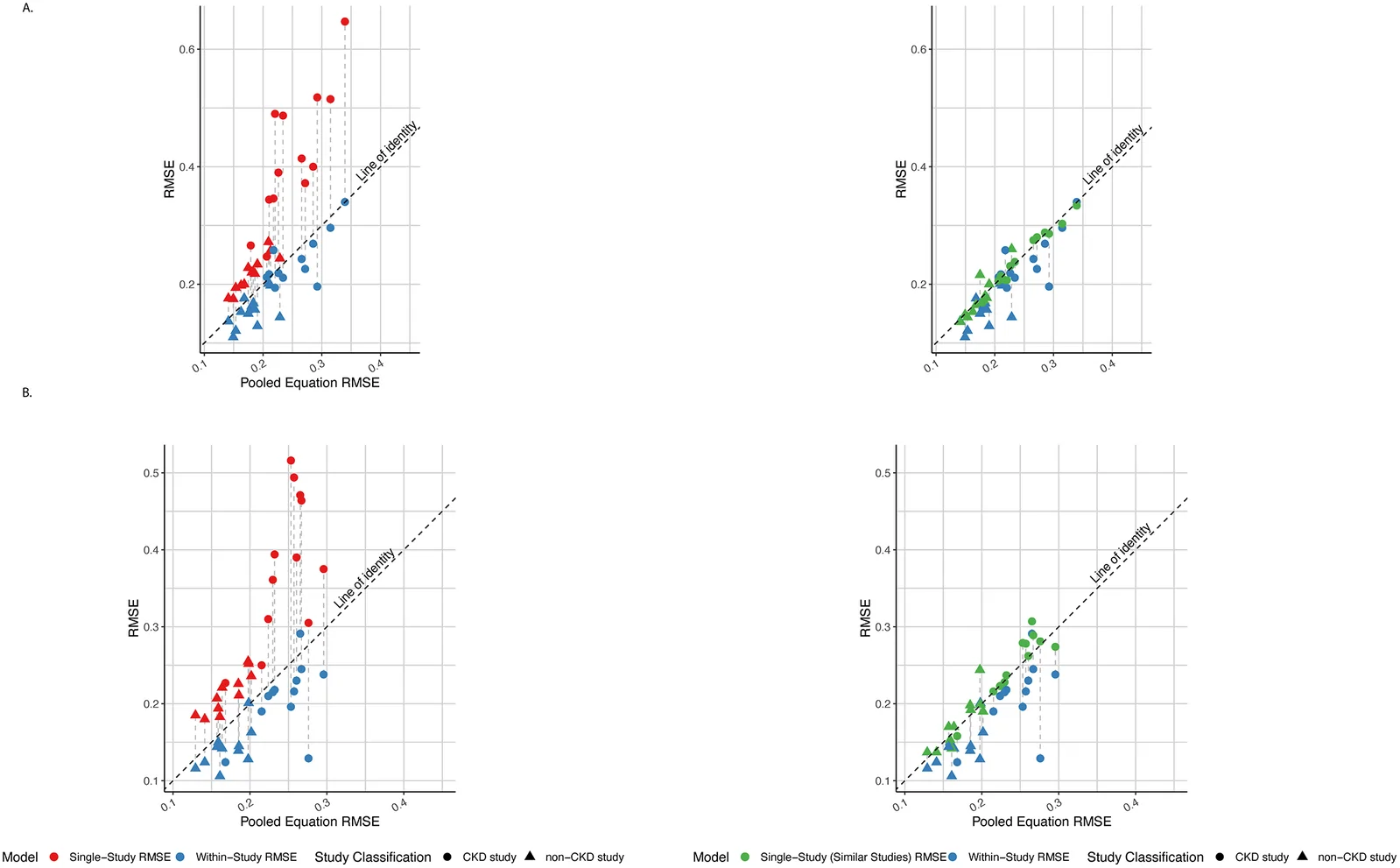
Comparison of within-study RMSE to single-study RMSE. The x-axis shows the average RMSE when predicting a given study using a model trained on all other studies combined (Pooled RMSE), and the y-axes show the average RMSE when predicting a given study when using single-study equations overall, when using within study equations, and when using single-study equations from within the same study classification (CKD vs. non-CKD study). Points above the dashed 1:1 line indicate that a given model has higher error than a pooled equation. Points are colored by the study classification (CKD study vs. non-CKD study). Panel A shows creatinine-based models while Panel B shows cystatin C-based models.

The mean RMSEs for single‐study equations exceeded within‐study RMSEs by factors ranging from approximately 1.3 up to 8.5 (overall mean ratio = 3.08, Table S8 in S2 File). Restricting single‐study equations to those the same study classification (i.e., same CKD classification) reduced this inflation: ratios fell to about 0.65 to 3.25 (overall mean ratio = 1.34), but they still did not match within‐study performance. Similar results were observed for cystatin C. These findings align with the error estimates from our fitted random-effects models. As shown in the Supplemental Methods, when a single-study equation is applied to a new study, the total prediction error is approximately the sum of the individual-level variation and coefficient error, plus about twice the study-level variation. For example, in a CKD female with creatinine of 2.0 mg/dL, the study-specific total variance was 0.012, compared to 0.044 for individual-level variation, meaning a single-study equation would underestimate error by nearly 50%.

## Discussion

CKD-EPI and other established GFR estimating equations were developed using pooled data from multiple studies to create versatile formulas for eGFR that are practical for clinical use. In our analyses, random effects models that explicitly allowed for study-level variation produced results consistent with the pooled equations, providing corroboration for the pooled data approach. At the same time, we revealed considerable variation in how measured GFR relates to creatinine, cystatin C, age, and sex across studies.

The apparent success of the pooled equations despite study-level heterogeneity likely reflects several features of the CKD-EPI development strategy. First, pooling across many studies averages over idiosyncratic recruitment patterns and measurement contexts, producing an equation that estimates the mean relationship expected across a broad target population. Second, the major structure of the relationship between filtration markers and mGFR is captured by the CKD-EPI functional form, so allowing random study effects primarily widens uncertainty and captures study-specific deviations rather than changing the population-average equation substantially. Third, pooling protects against overfitting to any single study. Thus, the same heterogeneity that creates uncertainty for individual prediction also explains why equations developed in one study may not transport well, whereas pooled equations can remain robust for broad clinical use.

Most of the variation across studies can be traced to systematic differences in how participants were recruited. Some studies enrolled individuals on the basis of disease status, such as patients with a clinical diagnosis of CKD or healthy kidney donor candidates, while others selected participants within specific ranges of creatinine or eGFRcr. These recruitment strategies created two broad classes of studies, which we have broadly described as CKD populations and non-CKD populations (even though some non-CKD populations included individuals with CKD), and much of the observed variation simply reflects difference between these study populations. This finding is consistent with the well recognized property predictive value of a diagnostic test depends on whether it is applied in a screening population or in patients with established disease. The change in the relationship between mGFR and its predictors depending on clinical context may seem like a useful insight to incorporate into equations for an individual patient; however, in practice clinicians often do not know whether a patient has CKD or how severe it is before ordering creatinine, and it is precisely in this setting of uncertainty that estimating equations are most commonly applied. The CKD-EPI equation, for instance, yields intermediate estimates of GFR between CKD and non-CKD populations, largely because non-CKD studies excluded participants with lower GFR, whereas CKD studies excluded those with higher GFR. Similar issues would be expected to arise for other GFR estimating equations developed from pooled populations, such as the EKFC equations [10,11].

As with all prediction equations, regression models for GFR estimates describe the average relationship between markers and mGFR with reasonable precision, but the uncertainty becomes much larger when applied to an individual. Our results provide quantification of the uncertainty in the eGFR. The magnitude of the uncertainty is consequential when eGFR values are close to decision thresholds. Small differences in eGFR may influence CKD staging, referral to nephrology, eligibility for therapies, medication dosing, or decisions to obtain confirmatory testing. In practice, this means that consistent with current clinical practice guidelines, an eGFR near a classification or referral threshold should be interpreted in clinical context, and confirmatory testing with cystatin C, combined creatinine-cystatin C eGFR, or measured GFR may be appropriate when the result would change management{, 2024 #185}. We were not able to incorporate all information that might be available to clinicians, such as extremes of muscle mass or diet. Prior work has not found that including common diseases or treatments as variables has improved the performance of creatinine-based eGFR equations [12]. Although “disease-specific” or “treatment-specific” eGFR equations could reduce variability in select populations, they may be impractical in clinical practice, as patients generally have more than one disease and undergo more than one treatment [13,14].

Our findings also illustrate why are estimating equations developed in a single study population are not recommended for application in widespread practice. When a GFR-estimating equation is developed within a single study, its reported error reflects primarily individual-level variation within that study and does not capture the study-to-study differences that arise when the equation is applied to new populations. Thus, internal validation can understate cross-study prediction error. Conversely, when an equation appears to perform differently at a new site, those differences may reflect expected study-to-study variation rather than a failure of the original equation.

Limitations of this analysis include our categorization of studies into CKD versus non-CKD was necessarily broad and encompassed heterogeneous subgroups. The non-CKD populations include healthy donor candidates as well as patients with HIV or diabetes who had high levels of GFR, while the CKD populations include CKD patients who have undergone transplant as well as those with disease in the native kidneys. We considered more granular classifications, including populations with CKD, populations at risk for CKD, and healthy populations, but selected the two-level classification to preserve model interpretability and focus on the primary distinction in study-entry criteria. Residual heterogeneity within these categories may contribute to the observed study-level differences. Importantly, some of these differences may reflect true biological variation, such as in transplant populations, where small changes in GFR have critical implications and warrant further investigation. In addition, we did not incorporate measurement error in creatinine or cystatin C, and our variance decomposition assumed a measurement-error variance of 0.015 for log mGFR, corresponding to an approximate CV of 12% based on variation roughly 4–16 weeks apart in large multi-center studies. This value was chosen a priori from repeated GFR measurements in two of the included studies. If the true measurement-error variance were smaller, for example closer to reports of median within-person CVs near 6% [15] based on repeat measurements in 40 patients over a mean 62 days apart, then a smaller proportion of total variation would be attributed to mGFR measurement error and a larger proportion would be attributed to within-study patient-level variability. The estimated between-study component, however, would be largely unchanged because it is determined by the random study effects rather than by the assumed mGFR measurement-error variance. Accounting for marker measurement error and refining study classifications could further clarify sources of heterogeneity.

Overall, our findings suggest that the CKD-EPI pooled equations are not subject to substantial residual confounding by study effects. Although measurable study-level heterogeneity exists, the pooled approach mitigates overfitting, accounts for study-to-study variation, and provides more reliable predictions than single-study equations. These results primarily provide empirical validation of the existing pooled CKD-EPI approach, while also quantifying the uncertainty that remains when any estimating equation is applied to an individual patient.

## Supporting information

S1 File Study Collaborators. Information on the data used in the paper.
(DOCX)

S2 File Supplementary Methods and Results. Additional information on the statistical methods as well as supplementary tables and figures.
(DOCX)
