# Supplementary material for: Study variation in estimating Glomerular Filtration Rate (GFR) from serum creatinine, cystatin C, age, and sex
Source: PLoS One. 2026 Sep 28;21(9):e0357209. doi: 10.1371/journal.pone.0357209 (PMC13618964; doi:10.1371/journal.pone.0357209)
Supplement: Supplementary file 1 [file pone.0357209.s001.docx]

# Study Collaborators

Data used for this paper is available from study personnel upon request.

**African American Study of Kidney Disease and Hypertension (AASK):** Tom Greene [Tom.Greene@hsc.utah.edu](mailto:Tom.Greene@hsc.utah.edu)

**The Age, Gene/Environment Susceptibility Reykjavik study (AGES-RS):** Vilmundur Gudnason [v.gudnason@hjarta.is](mailto:v.gudnason@hjarta.is)

**Assessing Long Term Outcomes in Living Kidney Donors (ALTOLD):** Roberto Kalil [RKalil@som.umaryland.edu](mailto:RKalil@som.umaryland.edu)

**Chronic Renal Insufficiency Cohort (CRIC):** Peter Wei Yang [weiyang@pennmedicine.upenn.edu](mailto:weiyang@pennmedicine.upenn.edu)

**Consortium for Radiologic Imaging Studies of Polycystic Kidney Disease (CRISP**): Vicente Torres [Torres.Vicente@mayo.edu](mailto:Torres.Vicente@mayo.edu)

**Captopril in Diabetic Nephropathy Study (CSG):** Roger A. Rodby [Roger_A_Rodby@rush.edu](mailto:Roger_A_Rodby@rush.edu)

**Diabetes Control and Complications Trial (DCCT):** Amy Karger [karge026@umn.edu](mailto:karge026@umn.edu)

**Study of people living with HIV:** Lesley Inker [Lesley.Inker@tuftsmedicine.org](mailto:Lesley.Inker@tuftsmedicine.org)

**Multicenter AIDS Cohort Study (MACS), now the MACS/WIHS Combined Cohort Study (MWCCS):** Michelle Estrella [Michelle.Estrella@ucsf.edu](mailto:Michelle.Estrella@ucsf.edu)

**Mayo Clinic:** John C. Lieske [Lieske.John@mayo.edu](mailto:Lieske.John@mayo.edu)

**Cleveland Clinic Foundation:** Emilio Poggio [poggioe@ccf.org](mailto:poggioe@ccf.org)

**Lund University:** Anders Grubb [anders.grubb@med.lu.se](mailto:anders.grubb@med.lu.se)

**Multi-Ethnic Study of Atherosclerosis (MESA):** Michael Shlipak [Michael.Shlipak@ucsf.edu](mailto:Michael.Shlipak@ucsf.edu)

**NephroTest:** Marc Froissart [marc.froissart@chuv.ch](mailto:marc.froissart@chuv.ch)

**Prevent Kidney Function Loss (PERL):** Alessandro Doria [Alessandro.Doria@joslin.harvard.edu](mailto:Alessandro.Doria@joslin.harvard.edu)

**Rein Angiotensin System Study (RASS):** Michael Mauer [mauer002@umn.edu](mailto:mauer002@umn.edu)

**Steno Diabetes Center study:** Peter Rossing [peter.rossing@regionh.dk](mailto:peter.rossing@regionh.dk)

**Diabetes Control and Complications Trial (DRDS):** Robert G. Nelson [rnelson@phx.niddk.nih.gov](mailto:rnelson@phx.niddk.nih.gov)

**University of Minnesota:** Jesse Seegmiller [jseegmil@umn.edu](mailto:jseegmil@umn.edu)

**University of Groningen:** Martin de Borst [m.h.de.borst@umcg.nl](mailto:m.h.de.borst@umcg.nl)
