# Supplementary material for: Study variation in estimating Glomerular Filtration Rate (GFR) from serum creatinine, cystatin C, age, and sex
Source: PLoS One. 2026 Sep 28;21(9):e0357209. doi: 10.1371/journal.pone.0357209 (PMC13618964; doi:10.1371/journal.pone.0357209)
Supplement: Supplementary file 2 [file pone.0357209.s002.docx]

# Supplemental Methods and Results

Contents

[1](#_Toc239158824)

[Supplemental Methods	2](#_Toc239158825)

[Supplemental Figures and Tables	6](#_Toc239158826)

# Supplemental Methods

A. Laboratory methods

We calibrated our measured serum creatinine using the Roche enzymatic method with the Roche-Hitachi P-Module instrument and Roche Creatininase Plus assay (Hoffman-La Roche, Ltd., Basel, Switzerland)^1^. This method is traceable to standards provided by the National Institute of Standards and Technology (NIST). Similarly, we calibrated our measured serum cystatin C using the Siemens Dade Behring Nephelometer, which is traceable to the International Federation for Clinical Chemists (IFCC) Working Group for the Standardization of Serum Cystatin C and the Institute for Reference Materials and Measurements (IRMM) certified reference materials^2,3^.

B. Statistical methods

*Random Effects Models*

The general structure of the CKD-EPI equations are given by

$$\begin{aligned} \log\left( mGFR \right)=\log\mu+\alpha_{1}\log\left\{ \min\left( \frac{Scr}{\kappa},1 \right) \right\}+\alpha_{2}\log\left\{ \max\left( \frac{Scr}{\kappa},1 \right) \right\}+\#\left( 1 \right) \end{aligned}$$

$$\beta_{1}\log\left\{ \min\left( \frac{Scys}{0.8},1 \right) \right\}+\beta_{2}\log\left\{ \max\left( \frac{Scys}{0.8},1 \right) \right\}+$$

$$\left\{ \log\left( \lambda\right)\times Age \right\}+\left\{ \log\left( \psi\right)\times Female \right\}+\epsilon$$

In equation 1, log 𝜇 is the intercept, $\alpha_{1}$ and $\alpha_{2}$ are the coefficients for log creatinine (Scr) below and above the creatinine knot point $\kappa$, $\beta_{1}$ and $\beta_{2}$ are the coefficients for log cystatin C (Scys) below and above the cystatin C knot point 0.8, and $\log\left( \lambda\right)$ and $\log\left( \psi\right)$are the coefficients for age and female sex, respectively. Female is equal to 1 if an individual is female and is 0 otherwise. The knot point 𝜅 for

creatinine is 0.7 mg/dL if an individual is female and is 0.9 mg/dL if male. The CKD-EPI creatinine model (eGFRcr) sets coefficients for cystatin C ($\beta_{1}$ and $\beta_{2}$) to zero, while the CKD-EPI cystatin C model (eGFRcys) sets coefficients for creatinine ($\alpha_{1}$ and $\alpha_{2}$) to zero. All coefficients are included in the CKD-EPI creatinine-cystatin C model (eGFRcr-cys).

The basic form of the random effects model is given by

$$\log\left( mGFR \right)=\left[ \mathrm{population}\mathrm{equation} \right]+\left[ deviation in the population equation for non-CKD studies vs. CKD studies \right]+\left[ unpredictable study deviations \right]+\left[ error in predicting true GFR within a given study \right]+[error as a result of measurement error in mGFR]$$

Specifically, for creatinine-based estimates, this model is represented by

$$\begin{aligned} \log\left( {mGFR}_{ij} \right)=\log\mu+\alpha_{1}\log\left\{ \min\left( \frac{{Scr}_{ij}}{\kappa},1 \right) \right\}+\alpha_{2}\log\left\{ \max\left( \frac{{Scr}_{ij}}{\kappa},1 \right) \right\}+\#\left( 2 \right) \end{aligned}$$

$$\left\{ \log\left( \lambda\right) \times{Age}_{ij} \right\}+\left\{ \log\left( \psi\right) \times{Female}_{ij} \right\}+$$

$$\log\tau Non-{CKD}_{j}+$$

$$\gamma_{1}\log\left\{ \min\left( \frac{{Scr}_{ij}}{\kappa},1 \right) \right\}*Non-{CKD}_{j}+\gamma_{2}\log\left\{ \max\left( \frac{{Scr}_{ij}}{\kappa},1 \right) \right\}*Non-{CKD}_{j}+$$

$$\begin{aligned} b_{0j}+b_{1j}\left[ log\left( {Scr}_{ij} \right)-log\left( \bar{Scr}_{j} \right) \right]+ \\ b_{3j}\left[ {Age}_{ij}-\bar{Age}_{j} \right]+b_{4j}\left[ {Female}_{ij}-\bar{Female}_{j} \right]+ \\ \epsilon_{ij\left( true \right)}+\epsilon_{ij\left( error \right)}\# \end{aligned}$$

For cystatin C-based estimates, the random effects model follows similarly,

$$\begin{aligned} \log\left( {mGFR}_{ij} \right)=\log\mu+\beta_{1}\log\left\{ \min\left( \frac{Scys}{0.8},1 \right) \right\}+\beta_{2}\log\left\{ \max\left( \frac{Scys}{0.8},1 \right) \right\}+\#\left( 3 \right) \end{aligned}$$

$$\left\{ \log\left( \lambda\right) \times{Age}_{ij} \right\}+\left\{ \log\left( \psi\right) \times{Female}_{ij} \right\}+$$

$$\log\tau Non-{CKD}_{j}+$$

$$\theta_{1}\log\left\{ \min\left( \frac{Scys}{0.8},1 \right) \right\}*Non-{CKD}_{j}+\theta_{2}\log\left\{ \max\left( \frac{Scys}{0.8},1 \right) \right\}*Non-{CKD}_{j}+$$

$$\begin{aligned} b_{0j}+b_{2j}\left[ log\left( {Scys}_{ij} \right)-log\left( \bar{Scys}_{j} \right) \right]+ \\ b_{3j}\left[ {Age}_{ij}-\bar{Age}_{j} \right]+b_{4j}\left[ {Female}_{ij}-\bar{Female}_{j} \right]+ \\ \epsilon_{ij\left( true \right)}+\epsilon_{ij\left( error \right)}\# \end{aligned}$$

In equations 2 and 3 above, ${mGFR}_{ij}$ denotes the mGFR for the $i$th individual in the $j$th study. The population equation for CKD studies is characterized by the intercept log 𝜇, the coefficients $\alpha_{1}$ and $\alpha_{2}$ for log creatinine (Scr) below and above the creatinine knot point $\kappa$, the coefficients $\beta_{1}$ and $\beta_{2}$ for log cystatin C below and above the cystatin C knot point $0.8$, and the coefficients for age and female sex $\log\left( \lambda\right)$ and $\log\left( \psi\right)$, respectively. $Non-{CKD}_{j}$is equal to 1 if a given study $j$ is a study of a non-CKD population, and 0 otherwise.

$\log\tau$ is the deviation in the intercept by the study classification. $\gamma_{1}$and$\gamma_{2}$are the coefficients that represent the deviations in the population creatinine terms by the study classification, while $\theta_{1}$ and $\theta_{2}$ are the coefficients that represent the deviations in the cystatin C terms by the study classification. $\bar{Scr}_{j}$, $\bar{Scys}_{j},$and $\bar{Age}_{j}$ are the mean creatinine, cystatin C, and age levels in study $j$ and $\bar{Female}_{j}$ is the fraction of females in study $j$. $b_{0j}$, $b_{1j}, b_{2j}$, $b_{3j}$and $b_{4j}$ are modeled as random effects and represent random study-specific deviations from the population-level fixed effects for the intercept and slopes for creatinine, cystatin C, age, and sex. Random effects were centered for interpretability and to reduce collinearity among them. We included only a single random slope for either creatinine or cystatin C, rather than modeling the knots, because within most studies, the distribution of these variables predominantly falls either above or below the given knot point. This single random slope approach allows for a study-specific tilt to be applied uniformly to all creatinine or cystatin C coefficients.

$\epsilon_{ij\left( true \right)}$ denotes error in estimating true GFR from the predictor variables for $i$th individual in the $j$th study, and $\epsilon_{ij\left( error \right)}$ denotes random measurement error in GFR. The $\epsilon_{ij\left( true \right)}$ are normally distributed with mean 0 and variance $\sigma_{true(CKD)}^{2}$, where the variance is a function of the CKD study classification such that CKD and non-CKD study populations can have different errors, while the $\epsilon_{ij\left( error \right)}$ are normally distributed with mean 0 and variance $\sigma_{error(CKD)}^{2}$. $\epsilon_{ij\left( true \right)}$ and $\epsilon_{ij\left( error \right)}$ are statistically independent.

The table below summarizes the statistical and clinical contexts for the terms in the creatinine based model (equation 2); the corresponding terms in the cystatin C based model (equation 3) can be interpreted similarly.

**Table**. Statistical and clinical contexts for the terms in equation 2.

| **Statistical Construct** | **Statistical Description** | **Clinical Interpretation** |
| --- | --- | --- |
| $\begin{aligned} \log\mu+\alpha_{1}\log\left\{ \min\left( \frac{{Scr}_{ij}}{\kappa},1 \right) \right\}+\alpha_{2}\log\left\{ \max\left( \frac{{Scr}_{ij}}{\kappa},1 \right) \right\}+ \end{aligned}$  $\left\{ \log\left( \lambda\right) \times{Age}_{ij} \right\}+\left\{ \log\left( \psi\right) \times{Female}_{ij} \right\}$ | Population mean GFR given Scr, sex and age, averaging across CKD studies | Mean GFR given Scr, sex and age across CKD studies |
| $\log\tau{non- CKD}_{j}+$  $\gamma_{1}\log\left\{ \min\left( \frac{{Scr}_{ij}}{\kappa},1 \right) \right\}*Non-{CKD}_{j}+\gamma_{2}\log\left\{ \max\left( \frac{{Scr}_{ij}}{\kappa},1 \right) \right\}*{Non-{CKD}_{j}}_{j}$ | Correction of the population mean GFR given Scr, sex and age, applied uniformly across the non-CKD studies | The impact of the context of measurement of SCR on the mean GFR given Scr, sex and age |
| $\begin{aligned} \log\mu+\alpha_{1}\log\left\{ \min\left( \frac{{Scr}_{ij}}{\kappa},1 \right) \right\}+\alpha_{2}\log\left\{ \max\left( \frac{{Scr}_{ij}}{\kappa},1 \right) \right\}+ \end{aligned}$  $\left\{ \log\left( \lambda\right) \times{Age}_{ij} \right\}+\left\{ \log\left( \psi\right) \times{Female}_{ij} \right\}$+ $\log\tau Non-{CKD}_{j}+$  $\gamma_{1}\log\left\{ \min\left( \frac{{Scr}_{ij}}{\kappa},1 \right) \right\}*Non-{CKD}_{j}+\gamma_{2}\log\left\{ \max\left( \frac{{Scr}_{ij}}{\kappa},1 \right) \right\}*Non-{CKD}_{j}$ | Population mean GFR given Scr, sex and age, averaging across non-CKD studies | Mean GFR given Scr, sex and age across non-CKD studies |
| $b_{0j}+b_{1j}\left[ log\left( {Scr}_{ij} \right)-log\left( \bar{Scr}_{j} \right) \right]+ b_{3j}\left[ {Age}_{ij}-\bar{Age}_{j} \right]+ b_{4j}\left[ {Female}_{ij}-\bar{Female}_{j} \right]$ | Correction to the population for $jth$ study | Impact of the specific context associated with the $jth$ study. |
| $\epsilon_{ij\left( true \right)}$ | Error in estimating true GFR from Scr, age and sex within the specific context of the $jth$ study. This error has mean 0 with variance $\sigma_{true(CKD)}^{2}$, where the variance is a function of the CKD study classification, such that CKD and non-CKD study populations can have different variances | Error in estimating true GFR from Scr, age and sex within the specific context of the $jth$ study |
| $\epsilon_{ij\left( error \right)}$ | Random measurement error in GFR. This error has mean 0 with variance $\sigma_{error}^{2}$ | Random measurement error in GFR |
| $b_{0j}+b_{1j}\left[ log\left( {Scr}_{ij} \right)-log\left( \bar{Scr}_{j} \right) \right]+ b_{3j}\left[ {Age}_{ij}-\bar{Age}_{j} \right]+ b_{4j}\left[ {Female}_{ij}-\bar{Female}_{j} \right]+ \epsilon_{ij\left( true \right)}$ | Error in estimating true GFR from Scr for a specific study, age and sex and accounting for CKD study classification | Error in estimating true GFR from Scr for a specific study, age and sex and accounting for CKD study classification |
| $b_{0j}+b_{1j}\left[ log\left( {Scr}_{ij} \right)-log\left( \bar{Scr}_{j} \right) \right]+ b_{3j}\left[ {Age}_{ij}-\bar{Age}_{j} \right]+ b_{4j}\left[ {Female}_{ij}-\bar{Female}_{j} \right]+ \epsilon_{ij\left( true \right)}+\epsilon_{ij\left( error \right)}$ | Error in estimating measured GFR from Scr for a specific study, age and sex and accounting for CKD study classification | Error in estimating measured GFR from Scr for a specific study, age and sex and accounting for CKD study classification |

*Error Estimates*

The total variance of $\log\left( GFR \right)$ estimates for the $i$th individual is given by

$$\mathrm{var}\left( {\log GFR}_{i} \right)=\underset{between-study variation}{\underbrace{Z_{i}^{'}GZ_{i}}}+ \underset{within-study variation}{\underbrace{\sigma_{true(CKD)}^{2}}}+\underset{measurement error}{\underbrace{\sigma_{error}^{2}}}$$

where $Z_{i}$ is the vector of covariates random effects terms from the $ith$ individual, $G$ is the unstructured $4\times4$ covariance matrix of the random effects (i.e., $G=Cov(b_{j})$), and $\sigma_{true}^{2}$ and $\sigma_{error}^{2}$ are defined as above. Thus, the total variance in log GFR can be partitioned in three parts: between-study variation, within-study variation, and measurement error in mGFR. The measurement error variance in log(mGFR) was externally estimated as 0.015, based on repeated GFR measurements over a 30-day interval in the African American Study of Kidney Disease and Hypertension (AASK) and the Modification of Diet in Renal Disease (MDRD) studies (**Table S2**). We report estimates of each variance component. Because variance–covariance structures in random-effects models depend on covariate values, we present estimates for several representative covariate levels. For example, in the creatinine-based model, the total variance in GFR for a female age 25 years with creatinine 2 mg/dL for a new CKD study that is 50% female, has a mean age of 50, and a mean creatinine of 1.7 mg/dL,

$$\mathrm{var}\left( \log GFR \right)=[1, \log\left( 2 \right)-log(1.7), 25-50, 1-0.5]G[1, \log\left( 2 \right)-log(1.7), 25-50, 1-0.5]'+ \sigma_{true}^{2}+\sigma_{error}^{2}$$

Next, we estimated the geometric mean GFR as a function of creatinine (or cystatin C) for males and females for individuals aged 50. Separate lines are given for CKD and non-CKD studies. Estimates are provided between the 5^th^ and 95^th^ percentile of creatinine (or cystatin C). We calculated the following four intervals around each line:

**Table.** Variance estimates for each interval

| Interval | Variance Estimate |
| --- | --- |
| 1. The confidence interval for mean mGFR of the population of studies | $X_{i}^{'}Cov(\hat{\beta})X_{i}$ |
| 2. The prediction intervals for within-study mean mGFR estimates | ${X_{i}^{'}Cov\left( \hat{\beta} \right)X_{i}+Z}_{i}^{'}GZ_{i}$ |
| 3. The prediction interval for true GFR at the patient level, given the fixed effects and accounting for both study and individual-level variation | $X_{i}^{'}Cov\left( \hat{\beta} \right)X_{i}+Z_{i}^{'}GZ_{i}+ \sigma_{true(CKD)}^{2}$ |
| 4. The prediction intervals for measured GFR at the patient level, given the fixed effects and accounting for both study and individual-level variation | $X_{i}^{'}Cov\left( \hat{\beta} \right)X_{i}+Z_{i}^{'}GZ_{i}+ \sigma_{true(CKD)}^{2}+\sigma_{error}^{2}$ |

Here, $X_{i}$ represents the covariates for the fixed-effect terms, and $Cov(\hat{\beta})$ is the covariance matrix of the fitted fixed effect terms. The intervals differ in their variability estimates but surround the same mean for the given CKD study classification.

*Total error in external applications when a single-study equation is applied in new populations*

Under the random effects model, the estimated mean measured GFR for an individual $i$ in study $k$ is given by$X_{ik}\hat{\beta}+Z_{ik}\hat{b}_{k}$.

Now suppose we apply this equation to an individual $j$ in study $l$. The true estimated log mGFR for this individual is $X_{jl}\beta+Z_{jl}b_{l}$. Hence, the prediction error for this new individual $j$ is

${X_{jl}\hat{\beta}+ Z}_{jl}\hat{b}_{k}$ - (${X_{jl}\beta+Z}_{jl}\hat{b}_{l}$ )$+ \epsilon_{ik\left( true \right)}+\epsilon_{ik\left( error \right)}={X_{jl}\left( \hat{\beta}-\beta\right)+Z}_{jl}(\hat{b}_{k}$ - $\hat{b}_{l})$ $+ \epsilon_{ik\left( true \right)}+\epsilon_{ik\left( error \right)}$

Because the random effects $b_{k}$and $b_{l}$ are independent, $var(b_{k}$- $b_{l})=$ $Z_{j}^{'}GZ_{j}{+Z}_{j}^{'}GZ_{j}=2*Z_{j}^{'}GZ_{j}$

Then, the total variance in new applications for individual$j$is given by

$$X_{j}^{'}Cov\left( \hat{\beta} \right)X_{j}+2*Z_{j}^{'}GZ_{j}+\sigma_{true(CKD)}^{2}+\sigma_{error}^{2}$$

Random effects models were fit in SAS Version 9.4 (Cary, NC) in *proc mixed* using restricted maximum likelihood (REML).

# Supplemental Figures and Tables

**Table S1**. Clinical Summary of the studies in CKD-EPI. Studies 1 through 13 denote studies of non-CKD populations and studies 14 through 26 studies of CKD populations. Studies are sorted by mean mGFR (ml/min/1.73m^2^).

| **Study #** | **n** | **Geography** | **Study Description** | **Entry Criteria as Reported** | **Method to measure GFR** |
| --- | --- | --- | --- | --- | --- |
| 1 | 250 | North America | RCT of type 1 diabetes with no diabetic nephropathy | Individuals with type 1 diabetes with no evidence of diabetic nephropathy | Urinary clearance of iothalamate and Plasma clearance of iohexol |
| 2 | 1176 | North America | RCT of type 1 diabetes | Individuals ages 13-39 with type 1 diabetes for 1 to 15 years, serum creatinine of 1.2 mg/dl, and normotensive by the standards used when the trial was initiated (140/90 mmHg) | Urinary clearance of iothalamate |
| 3 | 190 | North America | Cohort study of non-insulin-dependent diabetes mellitus in Pima Indians | Pima Indians with serum creatinine <1.3 mg/dL for women and <1.5 mg/dL for men | Urinary clearance of iothalamate |
| 4 | 457 | North America | Clinical population of kidney donors | Kidney donors over 18 years | Urinary clearance of iothalamate |
| 5 | 34 | Europe | Clinical population of kidney donors | Kidney donors | Urinary clearance of iothalamate |
| 6 | 96 | North America | Clinical population of kidney donors | Kidney donors over 18 years | Urinary clearance of iothalamate |
| 7 | 623 | North America | Cohort study of kidney donor candidates | Kidney donor candidates over 17 years with measures of serum creatinine | Urinary clearance of iothalamate |
| 8 | 164 | North America | Cohort study of kidney donors | Kidney donors | Plasma clearance of iohexol |
| 9 | 691 | North America | Cohort study of homosexual men | Homosexual and bisexual men | Plasma clearance of iohexol |
| 10 | 288 | North America | Clinical population of kidney donors | NR | Plasma clearance of iohexol |
| 11 | 200 | North America | Cross-sectional study of HIV | Individuals older than 18 years of age, on stable anti-retroviral for at least three months, with confirmed HIV status and an HIV viral load and CD4 count within 6 months of recruitment | Plasma clearance of iohexol |
| 12 | 294 | North America | General population of older adults | Adults aged 45-84 years with subclinical cardiovascular disease. Data included in our analysis was the subset of participants recruited into the MESA-Kidney ancillary study from 2012 to 2014 at John Hopkins University MESA field center in Baltimore | Plasma clearance of iohexol |
| 13 | 805 | Europe | Cohort study of aging related diseases | Surviving participants of the Reykjavik Study, a population-based study of individuals born in 1907–1935 and living in Reykjavik in 1966 | Plasma clearance of iohexol |
| 14 | 197 | North America | Cohort study of polycystic kidney disease | Individuals with polycystic kidney disease older than 15 years of age and younger than 46 years of age. Men were eligible if their serum creatinine concentrations were < 1.6 mg/dL and women were eligible if their serum creatinine concentrations were < 1.4 mg/dL. All subjects were required to have a 24-hour creatinine clearance > 70 mL/min/1.73 m^2^ or a Cockcroft Gault estimate of creatinine clearance > 70 mL/min to participate | Urinary clearance of iothalamate |
| 15 | 29 | Europe | Clinical population of kidney transplant recipients | Kidney transplant recipients | Urinary clearance of iothalamate |
| 16 | 684 | North America | RCT of insulin dependent diabetes | Patients 18 to 49 years of age were eligible if they had had insulin-dependent diabetes mellitus for at least 7 years, with an onset before the age of 30 years, and had diabetic retinopathy, urinary protein excretion of ≥ 500 mg per 24 hours, and a serum creatinine concentration of ≤ 2.5 mg per deciliter (221 μmol per liter) | Urinary clearance of iothalamate |
| 17 | 245 | Europe | RCT of type 1 diabetes with progressive diabetic nephropathy. | Individuals age 18 to 60 years with type 1 diabetes for at least ten years, diabetic retinopathy, albuminuria ≥ 300 mg/24hour and no evidence of kidney disease | Plasma clearance of Cr-EDTA |
| 18 | 489 | North America and Europe^1^ | RCT of type 1 diabetes at risk of high GFR loss | Individuals with type 1 diabetes, eGFR between 40 and 99.9 mL/min/1.73m2, SUA ≥ 4.5 mg/dL, and evidence of either a history of micro- or moderate macroalbuminuria during the prior 2 years or, in the absence of a history of albuminuria, 2) a rapid eGFR decline (≥3 mL/min/1.73 m2/year) before study enrollment | Plasma clearance of iohexol |
| 19 | 343 | Europe | Clinical population of individuals referred for GFR testing | Clinical population of individuals referred for mGFR testing | Plasma clearance of iohexol |
| 20 | 1807 | North America | RCT of CKD progression | African Americans aged 18 to 70 years with hypertensive renal disease (eGFR, 20-65 mL/min per 1.73 m2) | Urinary clearance of iothalamate |
| 21 | 88 | North America | Clinical population of CKD | Individuals over 18 with a clinical diagnosis of CKD | Urinary clearance of iothalamate |
| 22 | 669 | North America | Cohort study of chronic renal insufficiency | Patients aged 21 to 74 years with chronic renal insufficiency and meeting age-specific eGFR ranges | Urinary clearance of iothalamate |
| 23 | 521 | North America | Clinical population of CKD | Individuals with a clinical diagnosis of CKD (elevated serum creatinine level, proteinuria, abnormal urinary sediment, history of kidney disease, or kidney transplantation recipient) | Urinary clearance of iothalamate |
| 24 | 1701 | North America | RCT of CKD progression | Individuals age 18 to 70 and serum creatinine of 1.2 to 7.0 mg/dl in women and 1.4 to 7.0 mg/dl in men or a creatinine clearance of less than 70 ml/min/1.73m2 of body surface area, and a mean arterial pressure of 1.25 mm Hg or less | Urinary clearance of iothalamate |
| 25 | 313 | Europe | Clinical population of individuals referred for GFR testing | Individuals referred for mGFR testing | Plasma and urinary clearance of Cr-EDTA |
| 26 | 1037 | North America | Clinical population of CKD | Individuals over 18 with a clinical diagnosis of CKD |  |

Abbreviations: mGFR: measured Glomerular Filtration Rate; eGFR: estimated Glomerular Filtration Rate; BMI: Body Mass Index; NR: Not Reported;

**Table S2.** Estimation of measurement error variance in log(GFR) in MDRD and AASK

| **Mean GFR Range (ml/min/1.73m^2^)** | **MDRD Baseline^1^** | | **MDRD Follow-up^2^** | | **AASK Baseline^3^** | |
| --- | --- | --- | --- | --- | --- | --- |
|  | **N^4^** | **Mean Variance** | **N^4^** | **Mean Variance** | **N^4^** | **Mean Variance** |
| 15 – 25 | 249 | 0.0259 | 1090 | 0.0171 | 77 | 0.0149 |
| 25 – 35 | 266 | 0.0116 | 898 | 0.0119 | 192 | 0.0148 |
| 35 – 45 | 220 | 0.0124 | 729 | 0.0112 | 199 | 0.0165 |
| 45 – 55 | 185 | 0.0100 | 428 | 0.0102 | 264 | 0.0126 |
| 55 – 65 | 63 | 0.0064 | 77 | 0.0233 | 292 | 0.0111 |
| All | 1070 | 0.0155 | 3222 | 0.0135 | 1091 | 0.0141 |

^1^ Repeat GFRs over 30 day interval during baseline

^2^ Repeat GFRs over successive 4-month intervals starting at 4-months follow-up

^3^ Repeat GFRs over 1 – 6 week interval at baseline

^4^ Ns indicate number of pairs of GFR measurements used to compute the mean variances

Abbreviations: mGFR: measured Glomerular Filtration Rate; AASK: African American Study of Kidney Disease and Hypertension; MDRD: The Modification of Diet in Renal Disease study

**Table S3**. Characteristics of the CKD-EPI database, overall and by study. Studies 1 through 13 denote studies of non-CKD populations and studies 14 through 26 studies of CKD populations. Studies are sorted by mean mGFR (ml/min/1.73m^2^).

| **Study #** | **Sample Size** | **mGFR (ml/min/1.73m^2^)** | | **Creatinine (mg/dL)** | | **Cystatin C (mg/L)** | | | **eGFRcr (ml/min/**  **1.73m^2^)** | **eGFRcys (ml/min/**  **1.73m^2^)** | **eGFRcr-cys (ml/min/**  **1.73m^2^)** |
| --- | --- | --- | --- | --- | --- | --- | --- | --- | --- | --- | --- |
|  |  | **mean (SD)** | **2.5th%tle, 97.5%tle** | **mean (SD)** | **2.5th%tle, 97.5%tle** | **N with cystatin -c^†^** | **mean (SD)** | **2.5th%tle, 97.5%tle** | **mean (SD)** | **mean (SD)** | **mean (SD)** |
| Overall | | 68.4 (35.6) | 11.4, 139.0 | 1.5 (1.0) | 0.6, 4.5 | 7,496 | 1.2 (0.6) | 0.6, 3.0 | 71.0 (34.2) | 75.5 (32.9) | 78.7 (33.2) |
| 1 | Moderate (200–499) | 129.3 (18.9) | 95, 166 | 0.8 (0.1) | 0.5, 1 | 250 | 0.8 (0.1) | 0.6, 1 | 115.4 (13.5) | 113.8 (11.3) | 117.3 (11.9) |
| 2 | Very large (≥ 1,000) | 117.8 (19.5) | 84.6, 160.6 | 0.8 (0.1) | 0.5, 1 | 985 | 0.7 (0.1) | 0.6, 1 | 119.3 (10.7) | 118.2 (11.1) | 121.4 (10.1) |
| 3 | Small (50–199) | 112.3 (27.3) | 65.3, 165.1 | 0.7 (0.2) | 0.4, 1 | 0 | NR | NR | 114.7 (14.9) | NR | NR |
| 4 | Moderate (200–499) | 100.7 (17.5) | 68.8, 137.9 | 0.8 (0.2) | 0.5, 1.2 | 0 | NR | NR | 100.9 (14.3) | NR | NR |
| 5 | Very small (< 50) | 97.6 (18.3) | 69.4, 128.7 | 0.9 (0.1) | 0.7, 1.1 | 34 | 1.0 (0.1) | 0.8, 1.3 | 94.8 (15.1) | 83.0 (15.3) | 90.7 (14.3) |
| 6 | Small (50–199) | 97.0 (17.4) | 68.4, 134.4 | 0.8 (0.2) | 0.6, 1.1 | 96 | 0.8 (0.1) | 0.7, 1 | 103.2 (13.5) | 100.7 (13.8) | 104.8 (12.3) |
| 7 | Large (500–999) | 96.1 (15.6) | 68.4, 125.8 | 0.8 (0.2) | 0.5, 1.1 | 50 | 0.9 (0.1) | 0.7, 1.3 | 101.9 (14.6) | 89.8 (15.3) | 103.1 (13.7) |
| 8 | Small (50–199) | 96.1 (14.3) | 71, 123 | 0.8 (0.2) | 0.6, 1.1 | 164 | 0.8 (0.1) | 0.6, 1 | 102.7 (13.1) | 103.3 (14.7) | 106.4 (12.7) |
| 9 | Large (500–999) | 95.9 (21.1) | 52.8, 137.4 | 1.0 (0.3) | 0.7, 1.6 | 691 | 1.0 (0.3) | 0.7, 1.7 | 91.9 (18.2) | 86.3 (22.2) | 91.6 (20.0) |
| 10 | Moderate (200–499) | 93.8 (13.8) | 68.3, 122 | 0.8 (0.1) | 0.6, 1.1 | 288 | 0.8 (0.1) | 0.6, 1 | 105.1 (14.3) | 107.2 (14.2) | 109.4 (12.8) |
| 11 | Moderate (200–499) | 87.0 (25.7) | 39.1, 133.6 | 1.2 (0.4) | 0.7, 2 | 200 | 1.1 (0.4) | 0.6, 1.9 | 77.8 (22.5) | 82.3 (25.9) | 81.8 (23.5) |
| 12 | Moderate (200–499) | 72.6 (18.8) | 40.6, 108.7 | 0.9 (0.3) | 0.6, 1.5 | 294 | 1.0 (0.3) | 0.6, 1.8 | 79.2 (17.0) | 75.1 (22.5) | 80.8 (20.7) |
| 13 | Large (500–999) | 62.4 (16.5) | 28, 91 | 1.0 (0.4) | 0.6, 1.9 | 805 | 1.2 (0.4) | 0.8, 2.3 | 70.0 (17.8) | 60.9 (18.4) | 68.5 (19.1) |
| 14 | Small (50–199) | 90.5 (22.3) | 56, 135.2 | 0.9 (0.2) | 0.6, 1.4 | 197 | 0.9 (0.2) | 0.7, 1.4 | 96.6 (19.7) | 92.3 (18.1) | 95.4 (18.4) |
| 15 | Very small (< 50) | 77.5 (31.4) | 19.6, 129.9 | 1.1 (0.8) | 0.4, 2.9 | 29 | 1.4 (0.7) | 0.8, 3.1 | 87.6 (33.6) | 65.1 (30.5) | 75.4 (33.1) |
| 16 | Large (500–999) | 71.1 (30.7) | 18.3, 133.4 | 1.3 (0.5) | 0.7, 2.6 | 285 | 1.3 (0.6) | 0.7, 3 | 74.1 (27.0) | 70.6 (29.3) | 71.7 (29.2) |
| 17 | Moderate (200–499) | 68.2 (29.5) | 20, 126.3 | 1.4 (0.8) | 0.7, 3.4 | 245 | 1.4 (0.7) | 0.7, 2.9 | 74.2 (30.7) | 65.8 (30.4) | 70.2 (31.2) |
| 18 | Moderate (200–499) | 68.0 (17.0) | 38.5, 100.3 | 1.1 (0.3) | 0.7, 1.9 | 489 | 1.1 (0.3) | 0.7, 1.9 | 77.0 (19.6) | 72.4 (23.7) | 76.1 (22.0) |
| 19 | Moderate (200–499) | 63.6 (32.6) | 12, 122.9 | 1.5 (1.1) | 0.6, 4.5 | 343 | 1.6 (0.8) | 0.7, 3.9 | 65.1 (30.6) | 56.3 (29.1) | 61.2 (29.8) |
| 20 | Very large (≥ 1,000) | 54.4 (22.4) | 16.2, 98.7 | 1.7 (0.8) | 0.9, 4.1 | 833 | 1.5 (0.6) | 0.8, 3.1 | 51.8 (20.4) | 56.7 (24.5) | 54.7 (22.5) |
| 21 | Small (50–199) | 50.4 (29.6) | 9.7, 114 | 1.7 (1.1) | 0.6, 4.6 | 88 | 1.7 (0.9) | 0.8, 3.9 | 64.6 (35.3) | 52.4 (27.0) | 58.3 (30.9) |
| 22 | Large (500–999) | 47.1 (20.3) | 16.1, 94 | 1.7 (0.6) | 1, 3.2 | 314 | 1.6 (0.5) | 0.9, 2.8 | 45.8 (16.5) | 47.2 (19.5) | 46.7 (17.9) |
| 23 | Large (500–999) | 47.0 (25.5) | 11.4, 101.7 | 1.7 (1.0) | 0.6, 4.5 | 203 | 1.8 (0.8) | 0.9, 3.6 | 57.9 (29.8) | 48.0 (25.2) | 55.1 (28.8) |
| 24 | Very large (≥ 1,000) | 37.5 (20.1) | 10.4, 83.7 | 2.2 (1.1) | 0.9, 5 | 300 | 2.1 (0.7) | 1.1, 3.7 | 42.5 (21.2) | 34.3 (14.3) | 34.7 (14.7) |
| 25 | Moderate (200–499) | 33.6 (16.8) | 9.1, 70.9 | 2.4 (1.2) | 1.1, 5.8 | 313 | 2.0 (0.7) | 0.9, 3.6 | 34.6 (16.5) | 37.5 (19.1) | 36.3 (17.9) |
| 26 | Very large (≥ 1,000) | 32.8 (26.7) | 6.7, 99.8 | 2.9 (1.8) | 0.7, 7.2 | 0 | NR | NR | 37.1 (28.7) | NR | NR |
| Non-CKD populations (studies 1-13) | 5,268 | 96.6 (27.0) | 42.2, 150.0 | 0.9 (0.3) | 0.5, 1.5 | 5,268 | 0.9 (0.3) | 0.6, 1.8 | 98.6 (22.9) | 92.5 (27.8) | 97.1 (26.0) |
| CKD Populations (studies 14 -26) | 8,123 | 50.2 (27.7) | 10.4, 112.0 | 1.9 (1.2) | 0.7, 5.0 | 3,639 | 1.5 (0.8) | 0.7, 3.3 | 52.4 (27.6) | 56.9 (27.8) | 58.4 (28.2) |

**^†^** Cystatin C was available in only a subset of patients in some studies. All individuals have mGFR, creatinine, age, and sex measurements.

^*^ Within the PERL study, there were 16 total study sites: 15 in North America and 1 in Europe.

Abbreviations: mGFR: measured Glomerular Filtration Rate; eGFRcr: estimated Glomerular Filtration Rate based on creatinine; %tle: Percentile; BMI: Body Mass Index; NR: Not Reported.

**Table S4**. Demographic characteristics of the CKD-EPI database, overall and by study. Studies 1 through 13 denote studies of non-CKD populations and studies 14 through 26 studies of CKD populations.

| **Study #** | **Sample Size** | | **Black Race** | **Female Sex** | **Age** | **Diabetes*** | **BMI (kg/m^2^)** |
| --- | --- | --- | --- | --- | --- | --- | --- |
|  |  |  | **n (%)** | **n (%)** | **mean (SD)** | **n (%)** | **mean (SD)** |
| Overall | | | 3,250 (24.3) | 5,687 (42.4) | 49.6 (16.4) | 4,154 (32.0) | 27.7 (5.8) |
| 1 | Moderate (200–499) | | 6 ( 2.4) | 124 ( 49.6) | 31.3 (9.0) | 250 (100.0) | 25.7 (3.6) |
| 2 | Very large (≥ 1,000) | | 38 ( 3.2) | 541 ( 46.0) | 28.7 (5.7) | 1,176 (100.0) | 24.5 (3.0) |
| 3 | Small (50–199) | | 0 ( 0.0) | 110 (57.9) | 41.2 (10.9) | 159 (83.7) | 34.3 (8.0) |
| 4 | Moderate (200–499) | | 63 ( 13.8) | 280 ( 61.3) | 41.8 (10.1) | 0 ( 0.0) | 27.2 (4.3) |
| 5 | Very small (< 50) | | 0 ( 0.0) | 13 ( 38.2) | 51.9 (12.7) | 0 ( 0.0) | 26.3 (4.2) |
| 6 | Small (50–199) | | 10 ( 10.4) | 48 ( 50.0) | 42.9 (11.9) | 3 ( 3.1) | 26.4 (4.2) |
| 7 | Large (500–999) | | 6 ( 1.0) | 364 ( 58.4) | 41.4 (11.1) | 0 ( 0.0) | 27.9 (5.3) |
| 8 | Small (50–199) | | 4 ( 2.4) | 107 ( 65.2) | 44.1 (11.4) | 0 ( 0.0) | 26.7 (4.2) |
| 9 | Large (500–999) | | 235 (34.0) | 0 ( 0.0) | 52.5 (8.7) | 102 (18.6) | 26.4 (4.7) |
| 10 | Moderate (200–499) | | 10 ( 3.5) | 186 ( 64.6) | 40.4 (12.5) | NR | 26.6 (3.8) |
| 11 | Moderate (200–499) | | 104 (52.0) | 55 (27.5) | 47.8 (8.2) | 16 ( 8.0) | 26.6 (5.9) |
| 12 | Moderate (200–499) | | 139 (47.3) | 140 (47.6) | 70.7 (8.6) | 73 (24.8) | 29.7 (5.4) |
| 13 | Large (500–999) | | 0 ( 0.0) | 450 (55.9) | 80.0 (4.0) | 83 (10.3) | 27.3 (4.2) |
| 14 | Small (50–199) | | 21 (10.7) | 117 (59.4) | 33.5 (7.8) | 0 ( 0.0) | 26.1 (5.3) |
| 15 | Very small (< 50) | | 1 ( 3.4) | 17 (58.6) | 40.9 (14.4) | 5 (17.2) | 24.4 (3.3) |
| 16 | Large (500–999) | | 55 ( 8.0) | 312 ( 45.6) | 34.1 (7.7) | 684 (100.0) | 25.4 (4.8) |
| 17 | Moderate (200–499) | | 0 ( 0.0) | 85 ( 34.7) | 42.5 (9.1) | 245 (100.0) | 24.0 (3.3) |
| 18 | Moderate (200–499) | | 57 ( 11.7) | 163 ( 33.3) | 51.4 (11.0) | 489 (100.0) | 29.4 (5.9) |
| 19 | Moderate (200–499) | | 0 ( 0.0) | 167 (48.7) | 57.8 (15.6) | 66 (19.2) | 25.6 (5.5) |
| 20 | Very large (≥ 1,000) | | 1,807 (100.0) | 644 ( 35.6) | 53.8 (10.5) | 0 ( 0.0) | 30.6 (6.7) |
| 21 | Small (50–199) | | 7 ( 8.0) | 33 (37.5) | 53.7 (13.3) | 18 (20.5) | 28.4 (6.3) |
| 22 | Large (500–999) | | 289 (43.2) | 307 (45.9) | 54.7 (13.4) | 302 (45.1) | 31.7 (7.7) |
| 23 | Large (500–999) | | 1 ( 0.2) | 216 (41.5) | 54.0 (15.1) | 59 (11.3) | 29.1 (6.5) |
| 24 | Very large (≥ 1,000) | | 203 (11.9) | 671 (39.4) | 50.6 (12.7) | 103 ( 6.1) | 27.2 (4.7) |
| 25 | Moderate (200–499) | | 25 ( 8.0) | 90 (28.8) | 58.6 (14.7) | 72 (23.0) | 25.9 (4.4) |
| 26 | Very large (≥ 1,000) | | 169 (16.3) | 438 (42.2) | 54.3 (15.4) | 249 (24.0) | 28.4 (6.3) |
| Non-CKD populations (studies 1-13) | | 5,268 | 615 ( 11.7) | 2,418 (45.9) | 47.5 (19.3) | 1,862 (38.5) | 26.8 (4.9) |
| CKD Populations (studies 14 -26) | | 8,123 | 2,635 (332.4) | 3,269 (40.1) | 51.0 (14.1) | 2,292 (28.2) | 28.3 (6.2) |

*434 Ids are missing diabetes

Abbreviations: mGFR: measured Glomerular Filtration Rate; eGFRcr: estimated Glomerular Filtration Rate based on creatinine; %tle: Percentile; BMI: Body Mass Index; NR: Not Reported

**Table S5.** Study-specific effects regression models using creatinine (Panel A) or cystatin C (Panel B). Each row provides the estimated slopes from a regression line fit in each study separately. The gray row indicates the estimated slopes in the pooled 2021 CKD-EPI Creatinine equation. Studies 1 through 13 denote studies of non-CKD populations and studies 14 through 26 studies of CKD populations.

| **Study #** | **Sample Size** | **Coefficients for Creatinine (95% CI)** | | | **Coefficient** $\boldsymbol{c}$ **for Age,**  **per 1 year (95% CI)** | **Coefficient** $\boldsymbol{d}$**for Female Sex (95% CI)** | |
| --- | --- | --- | --- | --- | --- | --- | --- |
|  |  | **Slope for lower creatinine in males** | **Slope for lower creatinine in females** | **Slope for higher creatinine** |  |  |  |
| 2021 CKD-EPI Creatinine | | **-0.302 (-0.403, -0.202)** | **-0.241 (-0.344,**  **-0.138)** | **-1.200 (-1.211,**  **-1.189)** | **0.9938 (0.9935, 0.9942)** | **1.012 (1.000, 1.023)** | |
| 1 | Moderate (200–499) | -0.266 (-0.463,-0.068) | -0.266 (-0.463,-0.068) | -0.559 (-0.795,-0.323) | 0.997 (0.995,0.999) | 0.936 (0.898,0.975) | |
| 2 | Very large (≥ 1,000) | -0.314 (-0.429,-0.199) | -0.314 (-0.429,-0.199) | -0.350 (-0.549,-0.151) | 0.997 (0.995,0.999) | 1.014 (0.987,1.043) | |
| 3 | Small (50–199) | -0.343 (-0.593,-0.092) | -0.343 (-0.593,-0.092) | -0.904 (-1.307,-0.500) | 0.994 (0.992,0.997) | 0.890 (0.814,0.972) | |
| 4 | Moderate (200–499) | -0.338 (-0.655,-0.021) | -0.338 (-0.655,-0.021) | -0.435 (-0.589,-0.280) | 0.995 (0.993,0.996) | 1.059 (1.025,1.095) | |
| 5 | Very small (< 50) | -0.944 (-2.481,0.592) | -0.944 (-2.481,0.593) | -0.325 (-0.799,0.149) | 0.990 (0.986,0.994) | 1.001 (0.889,1.127) | |
| 6 | Small (50–199) | -1.097 (-1.795,-0.399) | -1.097 (-1.795,-0.399) | -0.174 (-0.565,0.217) | 0.993 (0.991,0.996) | 0.985 (0.912,1.063) | |
| 7 | Large (500–999) | -0.233 (-0.521,0.056) | -0.233 (-0.521,0.056) | -0.319 (-0.444,-0.193) | 0.995 (0.994,0.996) | 1.019 (0.993,1.046) | |
| 8 | Small (50–199) | -0.303 (-0.626,0.020) | -0.303 (-0.626,0.020) | -0.239 (-0.499,0.021) | 0.994 (0.993,0.996) | 0.975 (0.929,1.023) | |
| 9 | Large (500–999) | -0.447 (-0.605,-0.290) | -0.447 (-0.605,-0.290) | -0.760 (-0.837,-0.683) | 0.993 (0.991,0.994) | - | |
| 10 | Moderate (200–499) | -0.390 (-0.742,-0.038) | -0.390 (-0.742,-0.038) | -0.416 (-0.578,-0.255) | 0.994 (0.993,0.995) | 0.986 (0.954,1.019) | |
| 11 | Moderate (200–499) | -0.251 (-1.001,0.498) | -0.251 (-1.001,0.498) | -1.742 (-5.364,1.880) | 0.990 (0.987,0.994) | 0.940 (0.500,1.768) | |
| 12 | Moderate (200–499) | -0.444 (-0.745,-0.143) | -0.444 (-0.745,-0.143) | -0.880 (-0.992,-0.769) | 0.989 (0.987,0.991) | 0.994 (0.933,1.059) | |
| 13 | Large (500–999) | -0.425 (-0.687,-0.163) | -0.425 (-0.687,-0.163) | -0.864 (-0.959,-0.768) | 0.986 (0.983,0.988) | 0.887 (0.853,0.922) | |
| 14 | Small (50–199) | -0.232 (-1.013,0.549) | -0.232 (-1.013,0.549) | -0.952 (-0.995,-0.909) | 0.994 (0.990,0.997) | 0.962 (0.942,0.983) | |
| 15 | Very small (< 50) | -0.360 (-1.671,0.951) | -0.360 (-1.671,0.951) | -0.746 (-0.942,-0.550) | 0.994 (0.988,1.001) | 1.026 (0.967,1.089) | |
| 16 | Large (500–999) | 0.121 (-0.409,0.652) | 0.121 (-0.409,0.652) | -1.124 (-1.404,-0.844) | 0.995 (0.993,0.998) | 1.083 (0.881,1.331) | |
| 17 | Moderate (200–499) | -0.699 (-1.184,-0.215) | -0.699 (-1.184,-0.215) | -1.300 (-1.367,-1.234) | 0.994 (0.991,0.997) | 1.119 (1.071,1.169) | |
| 18 | Moderate (200–499) | -0.623 (-1.049,-0.196) | -0.623 (-1.049,-0.196) | -1.143 (-1.211,-1.076) | 0.994 (0.992,0.995) | 1.038 (0.980,1.100) | |
| 19 | Moderate (200–499) | 0.016 (-0.373,0.406) | 0.016 (-0.373,0.406) | -0.895 (-0.961,-0.828) | 0.994 (0.992,0.995) | 0.976 (0.947,1.007) | |
| 20 | Very large (≥ 1,000) | 0.120 (-1.082,1.323) | 0.120 (-1.082,1.323) | -1.197 (-1.250,-1.144) | 0.994 (0.993,0.996) | 1.057 (1.006,1.110) | |
| 21 | Small (50–199) | 0.059 (-0.445,0.563) | 0.059 (-0.445,0.563) | -1.076 (-1.104,-1.048) | 0.992 (0.989,0.996) | 0.968 (0.946,0.989) | |
| 22 | Large (500–999) |  | - | -1.099 (-1.193,-1.004) | 0.995 (0.993,0.996) | 0.928 (0.833,1.033) | |
| 23 | Large (500–999) | -0.171 (-0.596,0.255) | -0.171 (-0.596,0.255) | -1.217 (-1.281,-1.154) | 0.993 (0.991,0.994) | 1.038 (0.999,1.079) | |
| 24 | Very large (≥ 1,000) | -1.125 (-3.278,1.027) | -1.125 (-3.278,1.027) | -1.133 (-1.187,-1.079) | 0.995 (0.994,0.996) | 0.997 (0.949,1.048) | |
| 25 | Moderate (200–499) |  | - | -1.160 (-1.181,-1.139) | 0.995 (0.993,0.996) | 0.995 (0.976,1.015) | |
| 26 | Very large (≥ 1,000) | -0.519 (-1.541,0.503) | -0.519 (-1.541,0.503) | -1.119 (-1.180,-1.059) | 0.993 (0.992,0.995) | 0.923 (0.872,0.978) | |
| **Studies 1-13** | 5,268 | -0.299 (-0.363,-0.234) | -0.311 (-0.379,-0.243) | -0.828 (-0.859, -0.796) | 0.991 (0.991,0.991) | | 0.972 (0.961,0.983) |
| **Studies 14-26** | 8,123 | 0.063 (-0.120,0.247) | 0.063 (-0.184,0.310) | -1.156 (-1.167, -1.14) | 0.994 (0.994,0.995) | | 1.006 (0.994,1.0177) |

| **Study #** | **Sample Size** | **Coefficients for Cystatin C (95% CI)** | | **Coefficient** $\boldsymbol{c}$ **for Age,**  **per 1 year (95% CI)** | **Coefficient** $\boldsymbol{d}$**for Female Sex (95% CI)** |
| --- | --- | --- | --- | --- | --- |
|  |  | **Slope for lower values** | **Slope for higher values** |  |  |
| **2021 CKD-EPI Cystatin C** | | **-0.499 (-0.610, -0.388)** | **-1.328 (-1.344,**  **-1.312)** | **0.9962 (0.9957, 0.9966)** | **0.932 (0.921, 0.944)** |
| 1 | Moderate (200–499) | -0.752 (-0.962,-0.543) | -0.470 (-0.768,-0.172) | 0.997 (0.9948,0.9981) | 0.883 (0.855,0.912) |
| 2 | Very large (≥ 1,000) | -0.463 (-0.577,-0.350) | -0.395 (-0.619,-0.171) | 0.997 (0.9947,0.9983) | 0.959 (0.939,0.980) |
| 3 | Small (50–199) | - | - | - | - |
| 4 | Moderate (200–499) | - | - | - | - |
| 5 | Very small (< 50) | 0.009 (-2.411,2.428) | -0.491 (-0.915,-0.067) | 0.9915 (0.987,0.9957) | 0.948 (0.853,1.055) |
| 6 | Small (50–199) | -0.938 (-1.642,-0.235) | -0.620 (-0.995,-0.246) | 0.996 (0.994,0.9991) | 0.922 (0.862,0.986) |
| 7 | Large (500–999) | -0.892 (-1.894,0.110) | -0.651 (-0.958,-0.344) | 0.995 (0.991,0.9983) | 0.951 (0.876,1.032) |
| 8 | Small (50–199) | -0.101 (-0.307,0.106) | -0.653 (-0.933,-0.374) | 0.995 (0.994,0.9970) | 0.957 (0.918,0.998) |
| 9 | Large (500–999) | -0.134 (-0.394,0.126) | -0.711 (-0.772,-0.650) | 0.994 (0.993,0.9957) | - |
| 10 | Moderate (200–499) | -0.594 (-0.741,-0.446) | -0.580 (-0.784,-0.376) | 0.996 (0.995,0.9971) | 0.951 (0.926,0.976) |
| 11 | Moderate (200–499) | -0.405 (-0.911,0.101) | -0.872 (-0.990,-0.753) | 0.995 (0.992,0.9989) | 0.940 (0.882,1.002) |
| 12 | Moderate (200–499) | -0.456 (-0.749,-0.163) | -0.825 (-0.904,-0.747) | 0.995 (0.993,0.9973) | 0.865 (0.837,0.894) |
| 13 | Large (500–999) | -0.060 (-0.506,0.386) | -1.015 (-1.056,-0.974) | 0.994 (0.991,0.9966) | 0.936 (0.917,0.955) |
| 14 | Small (50–199) | 0.084 (-0.593,0.760) | -0.770 (-0.988,-0.553) | 0.991 (0.987,0.9947) | 0.967 (0.910,1.027) |
| 15 | Very small (< 50) | 1.814 (-3.090,6.717) | -1.201 (-1.413,-0.989) | 0.996 (0.990,1.0017) | 0.975 (0.834,1.139) |
| 16 | Large (500–999) | -0.664 (-1.869,0.541) | -1.343 (-1.472,-1.214) | 1.001 (0.993,1.0088) | 0.953 (0.868,1.046) |
| 17 | Moderate (200–499) | -0.624 (-1.282,0.034) | -1.235 (-1.297,-1.173) | 0.995 (0.993,0.9977) | 0.916 (0.872,0.962) |
| 18 | Moderate (200–499) | 0.050 (-0.239,0.338) | -0.884 (-0.931,-0.837) | 0.997 (0.996,0.9977) | 0.901 (0.880,0.923) |
| 19 | Moderate (200–499) | 0.114 (-0.654,0.883) | -1.360 (-1.426,-1.294) | 0.999 (0.997,1.0007) | 0.955 (0.907,1.007) |
| 20 | Very large (≥ 1,000) | -0.505 (-1.360,0.351) | -1.136 (-1.179,-1.093) | 0.998 (0.996,0.9993) | 0.923 (0.895,0.953) |
| 21 | Small (50–199) | 0.294 (-2.568,3.155) | -1.397 (-1.527,-1.267) | 0.994 (0.990,0.9981) | 0.884 (0.787,0.994) |
| 22 | Large (500–999) | 1.603 (-1.564,4.770) | -1.275 (-1.360,-1.191) | 0.998 (0.997,1.0004) | 0.926 (0.880,0.975) |
| 23 | Large (500–999) | - | -1.471 (-1.561,-1.382) | 0.999 (0.996,1.0008) | 1.009 (0.939,1.083) |
| 24 | Very large (≥ 1,000) | - | -1.284 (-1.362,-1.207) | 1.001 (0.999,1.0031) | 0.907 (0.861,0.955) |
| 25 | Moderate (200–499) | - | -1.273 (-1.346,-1.199) | 0.999 (0.998,1.0011) | 0.815 (0.769,0.865) |
| 26 | Very large (≥ 1,000) | - | - | - | - |
| **Studies 1-2, 5-13** | 3,857 | -0.318 (-0.391,-0.245) | -0.853 (-0.882,-0.824) | 0.994 (0.994,0.995) | 0.903 (0.893,0.913) |
| **Studies 14-25** | 3,639 | 0.071 (-0.205,0.346) | -1.293 (-1.313,-1.272) | 0.997 (0.996,0.998) | 0.935 (0.920,0.950) |

Cells marked with “–” indicate that the coefficient could not be estimated for that study. Study 9 included only male participants. For creatinine, studies 22 and 25 lacked sufficient data before the serum creatinine knot. For cystatin C, studies 3, 4, and 26 did not report cystatin C measurements, while studies 23, 24, and 25 did not have enough data before the cystatin C knot.

**Table S6A.** Estimated fixed effect regression coefficients from the random effects model with creatinine (model n=13,391). All coefficients are reported on the log scale.

| **Term** | **Coefficient (95%CI)** | **P** |
| --- | --- | --- |
| Intercept | 4.86 (4.80, 4.93) | <.0001 |
| Pre-knot slope for log creatinine in males | -0.14 (-0.33, 0.05) | 0.05 |
| Difference in pre-knot slope for creatinine for females vs. males | 0.004 (-0.29, 0.30) | 0.98 |
| Post-knot slope for log serum creatinine | -1.14 (-1.20, -1.07) | <.0001 |
| Deviation in the intercept for Non-CKD studies | 0.04 (-0.03, 0.11) | 0.23 |
| Deviation in pre-knot slope for log creatinine in males for non-CKD studies | -0.23 (-0.44, -0.02) | 0.03 |
| Deviation in the difference in pre-knot slope for log creatinine for females vs. males for non-CKD studies | -0.01 (-0.33, 0.30) | 0.93 |
| Deviation in post-knot slope for log creatinine for non-CKD studies | 0.52 (0.43, 0.61) | <.0001 |
| Female | -0.008 (-0.03, 0.01) | 0.44 |
| Age | -0.006 (-0.007, -0.006) | <.0001 |

**Table S6B.** Standard deviations of the random effects from the random effects model with creatinine**.**

| **Effect** | **Standard Deviation** |
| --- | --- |
| **Intercept** | 0.083 |
| **Log Creatinine** | 0.136 |
| **Female** | 0.062 |
| **Age** | 0.002 |

**Table S6C.** Correlation matrix of the random effects from the random effects model with creatinine.

| **Effect** | **Intercept** | **Log Scr** | **Female** | **Age** |
| --- | --- | --- | --- | --- |
| **Intercept** | 1.00 | 0.28 | 0.32 | 0.70 |
| **Log Creatinine** | 0.28 | 1.00 | 0.68 | 0.70 |
| **Female** | 0.32 | 0.68 | 1.00 | 0.70 |
| **Age** | 0.70 | 0.70 | 0.70 | 1.00 |

**Table S7A.** Estimated fixed effect regression coefficients from the random effects model with cystatin C (model n (n=7,496). All coefficients are reported on the log scale.

| **Term** | **Coefficient (95%CI)** | **P** |
| --- | --- | --- |
| Intercept | 4.81 (4.73, 4.89) | <.0001 |
| Pre-knot slope for log cystatin C | -0.11 (-0.39, 0.17) | 0.44 |
| Post-knot slope for log cystatin C | -1.19 (-1.27, -1.10) | <.0001 |
| Deviation in the intercept for non-CKD studies | 0.002 (-0.08, 0.09) | 0.95 |
| Deviation in pre-knot slope for log cystatin C for non-CKD studies | -0.37 (-0.67, -0.07) | 0.02 |
| Deviation in post-knot slope for log cystatin C for non-CKD studies | 0.37 (0.24, 0.50) | <.0001 |
| Female | -0.07 (-0.09, -0.05) | <.0001 |
| Age | -0.004 (-0.005, -0.003) | <.0001 |

**Table S7B.** Standard deviations of the random effects from the random effects model with cystatin C.

| **Effect** | **Standard Deviation** |
| --- | --- |
| **Intercept** | 0.100 |
| **Log cystatin C** | 0.162 |
| **Female** | 0.039 |
| **Age** | 0.002 |

**Table S7C.** Correlation matrix of the random effects from the random effects model with cystatin C.

| **Effect** | **Intercept** | **Log Cys** | **Female** | **Age** |
| --- | --- | --- | --- | --- |
| **Intercept** | 1.00 | 0.41 | 0.23 | -0.52 |
| **Log cystatin C** | 0.41 | 1.00 | 0.02 | -0.56 |
| **Female** | 0.23 | 0.02 | 1.00 | -0.21 |
| **Age** | -0.52 | -0.56 | -0.21 | 1.00 |

**Table S8.** Cross-Study RMSE Performance Metrics for models evaluated on a given study. Studies 1 through 13 denote studies of non-CKD populations and studies 14 through 26 studies of CKD populations. Panel A shows creatinine-based models while Panel B shows cystatin C-based models

| **Study #** | **Study Sample Size** | **A. Average RMSE from within-study cross‑validation** | **B. RMSE when predicting a given study using a model trained on all other studies combined** | **C. RMSE when predicting a given study using a model trained on all similar studies combined** | **D. RMSE using the fixed effect coefficients from random effects model*** | **E. Average RMSE when predicting given study when using single study equations** | **F. Average RMSE when predicting given study when using single study equations from similar studies** | **Ratio of average MSE from single-study equations to average MSE from within-study cross-validation** | **Ratio of average MSE from s single-study equations from similar studies to average MSE from within-study cross-validation from similar studies** |
| --- | --- | --- | --- | --- | --- | --- | --- | --- | --- |
| 1 | Moderate (200–499) | 0.129 | 0.190 | 0.177 | 0.201 | 0.234 | 0.200 | 3.518 | 2.400 |
| 2 | Very large (≥ 1,000) | 0.168 | 0.184 | 0.191 | 0.180 | 0.220 | 0.180 | 1.746 | 1.144 |
| 3 | Small (50–199) | 0.198 | 0.213 | 0.216 | 0.213 | 0.257 | 0.213 | 1.690 | 1.160 |
| 4 | Moderate (200–499) | 0.157 | 0.186 | 0.187 | 0.177 | 0.218 | 0.177 | 2.007 | 1.269 |
| 5 | Very small (< 50) | 0.121 | 0.154 | 0.151 | 0.145 | 0.194 | 0.144 | 2.237 | 1.415 |
| 6 | Small (50–199) | 0.176 | 0.168 | 0.173 | 0.165 | 0.200 | 0.165 | 1.288 | 0.879 |
| 7 | Large (500–999) | 0.153 | 0.163 | 0.166 | 0.154 | 0.198 | 0.153 | 1.729 | 1.003 |
| 8 | Small (50–199) | 0.137 | 0.141 | 0.144 | 0.136 | 0.176 | 0.136 | 1.693 | 0.990 |
| 9 | Large (500–999) | 0.163 | 0.181 | 0.181 | 0.173 | 0.220 | 0.173 | 1.855 | 1.130 |
| 10 | Moderate (200–499) | 0.110 | 0.149 | 0.162 | 0.148 | 0.175 | 0.148 | 2.691 | 1.809 |
| 11 | Moderate (200–499) | 0.201 | 0.209 | 0.202 | 0.208 | 0.272 | 0.210 | 1.863 | 1.089 |
| 12 | Moderate (200–499) | 0.150 | 0.175 | 0.163 | 0.214 | 0.228 | 0.216 | 2.495 | 2.065 |
| 13 | Large (500–999) | 0.144 | 0.229 | 0.203 | 0.257 | 0.244 | 0.260 | 3.310 | 3.254 |
| 14 | Small (50–199) | 0.212 | 0.206 | 0.203 | 0.206 | 0.247 | 0.206 | 1.332 | 0.946 |
| 15 | Very small (< 50) | 0.258 | 0.218 | 0.212 | 0.207 | 0.346 | 0.207 | 1.480 | 0.646 |
| 16 | Large (500–999) | 0.269 | 0.285 | 0.285 | 0.289 | 0.400 | 0.288 | 2.336 | 1.149 |
| 17 | Moderate (200–499) | 0.217 | 0.210 | 0.211 | 0.214 | 0.344 | 0.214 | 2.659 | 0.968 |
| 18 | Moderate (200–499) | 0.157 | 0.179 | 0.180 | 0.169 | 0.266 | 0.169 | 3.167 | 1.158 |
| 19 | Moderate (200–499) | 0.219 | 0.226 | 0.224 | 0.231 | 0.390 | 0.231 | 3.723 | 1.109 |
| 20 | Very large (≥ 1,000) | 0.226 | 0.272 | 0.275 | 0.281 | 0.372 | 0.280 | 3.067 | 1.536 |
| 21 | Small (50–199) | 0.196 | 0.292 | 0.297 | 0.286 | 0.518 | 0.286 | 7.442 | 2.126 |
| 22 | Large (500–999) | 0.243 | 0.266 | 0.263 | 0.276 | 0.414 | 0.275 | 3.343 | 1.281 |
| 23 | Large (500–999) | 0.296 | 0.315 | 0.319 | 0.302 | 0.515 | 0.303 | 3.497 | 1.045 |
| 24 | Very large (≥ 1,000) | 0.194 | 0.220 | 0.217 | 0.207 | 0.490 | 0.207 | 8.525 | 1.144 |
| 25 | Moderate (200–499) | 0.211 | 0.234 | 0.231 | 0.239 | 0.487 | 0.238 | 6.941 | 1.275 |
| 26 | Very large (≥ 1,000) | 0.340 | 0.340 | 0.340 | 0.334 | 0.647 | 0.334 | 4.495 | 0.967 |
| **Mean, Studies 1-13** | 5,268 | 0.154 | 0.180 | 0.178 | 0.182 | 0.218 | 0.183 | 2.163 | 1.508 |
| **Mean, Studies 14-26** | 8,123 | 0.234 | 0.251 | 0.251 | 0.249 | 0.418 | 0.249 | 4.001 | 1.181 |
| **Overall** | 13,391 | 0.194 | 0.216 | 0.214 | 0.216 | 0.318 | 0.216 | 3.082 | 1.344 |

| **Study #** | **Study Sample Size** | **A.** **Average RMSE from within-study cross‑validation** | **B. RMSE** **when predicting a given study using a model trained on all other studies combined** | **C. RMSE when predicting a given study using a model trained on all similar studies combined** | **D. RMSE using the fixed effect coefficients from random effects model*** | **E. Average RMSE when predicting given study when using single study equations** | **F. Average RMSE when predicting given study when using single study equations from similar studies** | **Ratio of average MSE from single-study equations to average MSE from within-study cross-validation** | **Ratio of average MSE from s single-study equations from similar studies to average MSE from within-study cross-validation from similar studies** |
| --- | --- | --- | --- | --- | --- | --- | --- | --- | --- |
| 1 | Moderate (200–499) | 0.128 | 0.198 | 0.188 | 0.241 | 0.255 | 0.244 | 4.412 | 3.627 |
| 2 | Very large (≥ 1,000) | 0.142 | 0.164 | 0.168 | 0.170 | 0.221 | 0.170 | 2.639 | 1.438 |
| 3 | Small (50–199) | - | - |  | - | - | - | - | - |
| 4 | Moderate (200–499) | - | - |  | - | - | - | - | - |
| 5 | Very small (< 50) | 0.139 | 0.185 | 0.179 | 0.196 | 0.226 | 0.198 | 2.599 | 2.020 |
| 6 | Small (50–199) | 0.150 | 0.159 | 0.164 | 0.153 | 0.194 | 0.153 | 1.574 | 1.042 |
| 7 | Large (500–999) | 0.116 | 0.129 | 0.127 | 0.137 | 0.185 | 0.137 | 2.601 | 1.404 |
| 8 | Small (50–199) | 0.124 | 0.141 | 0.141 | 0.137 | 0.180 | 0.137 | 2.091 | 1.215 |
| 9 | Large (500–999) | 0.163 | 0.202 | 0.195 | 0.189 | 0.236 | 0.190 | 2.129 | 1.353 |
| 10 | Moderate (200–499) | 0.106 | 0.161 | 0.171 | 0.143 | 0.183 | 0.142 | 3.109 | 1.796 |
| 11 | Moderate (200–499) | 0.201 | 0.198 | 0.200 | 0.199 | 0.252 | 0.199 | 1.560 | 0.977 |
| 12 | Moderate (200–499) | 0.144 | 0.157 | 0.149 | 0.172 | 0.207 | 0.170 | 2.162 | 1.400 |
| 13 | Large (500–999) | 0.145 | 0.186 | 0.168 | 0.195 | 0.211 | 0.192 | 2.285 | 1.747 |
| 14 | Small (50–199) | 0.190 | 0.215 | 0.222 | 0.216 | 0.250 | 0.216 | 1.739 | 1.288 |
| 15 | Very small (< 50) | 0.129 | 0.276 | 0.289 | 0.283 | 0.305 | 0.281 | 4.191 | 4.738 |
| 16 | Large (500–999) | 0.230 | 0.261 | 0.253 | 0.262 | 0.390 | 0.262 | 2.894 | 1.302 |
| 17 | Moderate (200–499) | 0.210 | 0.224 | 0.239 | 0.225 | 0.310 | 0.223 | 2.048 | 1.131 |
| 18 | Moderate (200–499) | 0.124 | 0.168 | 0.172 | 0.157 | 0.227 | 0.158 | 3.698 | 1.628 |
| 19 | Moderate (200–499) | 0.238 | 0.296 | 0.286 | 0.275 | 0.375 | 0.274 | 2.647 | 1.326 |
| 20 | Very large (≥ 1,000) | 0.215 | 0.230 | 0.233 | 0.227 | 0.361 | 0.228 | 3.153 | 1.120 |
| 21 | Small (50–199) | 0.291 | 0.265 | 0.270 | 0.308 | 0.471 | 0.307 | 2.875 | 1.112 |
| 22 | Large (500–999) | 0.218 | 0.232 | 0.229 | 0.237 | 0.394 | 0.237 | 3.741 | 1.183 |
| 23 | Large (500–999) | 0.245 | 0.267 | 0.265 | 0.289 | 0.464 | 0.289 | 4.076 | 1.395 |
| 24 | Very large (≥ 1,000) | 0.196 | 0.253 | 0.245 | 0.278 | 0.516 | 0.279 | 8.523 | 2.021 |
| 25 | Moderate (200–499) | 0.216 | 0.257 | 0.256 | 0.279 | 0.494 | 0.278 | 6.159 | 1.660 |
| 26 | Very large (≥ 1,000) | - | - | - | - | - | - | - | - |
| **Mean, Studies 1-2, 5-13** | 5,268 | 0.142 | 0.171 | 0.154 | 0.176 | 0.214 | 0.176 | 2.469 | 1.638 |
| **Mean, Studies 14-25** | 3,639 | 0.209 | 0.240 | 0.247 | 0.253 | 0.380 | 0.253 | 3.812 | 1.659 |
| **Overall** | 8,907 | 0.177 | 0.210 | 0.209 | 0.216 | 0.300 | 0.216 | 3.170 | 1.649 |

Studies 3, 4, and 26 did not report cystatin C measurements.

References

1.	Levey AS, Coresh J, Greene T, et al. Expressing the Modification of Diet in Renal Disease Study equation for estimating glomerular filtration rate with standardized serum creatinine values. *Clin Chem*. Apr 2007;53(4):766–72. doi:10.1373/clinchem.2006.077180

2.	Grubb A, Blirup-Jensen S, Lindström V, Schmidt C, Althaus H, Zegers I. First certified reference material for cystatin C in human serum ERM-DA471/IFCC. *Clin Chem Lab Med*. Nov 2010;48(11):1619–21. doi:10.1515/cclm.2010.318

3.	Blirup-Jensen S, Grubb A, Lindstrom V, Schmidt C, Althaus H. Standardization of Cystatin C: development of primary and secondary reference preparations. *Scand J Clin Lab Invest Suppl*. 2008;241:67–70. doi:10.1080/00365510802150067
