# Supplementary material for: Study variation in estimating Glomerular Filtration Rate (GFR) from serum creatinine, cystatin C, age, and sex
Source: PLoS One. 2026 Sep 28;21(9):e0357209. doi: 10.1371/journal.pone.0357209 (PMC13618964; doi:10.1371/journal.pone.0357209)
Supplement: Supplementary file 3 [file pone.0357209.s004.docx]

Response to Reviewers

Thank you very much for reviewing our paper and providing feedback that has strengthend our paper. Below are our responses to specific points raised by the reviewers.

**Reviewer #1:** interesting study that examines sources of variation between mGFR and eGFR; found differing sources of variation (study variation, measurement error, patient -level errors) in non CKD and CKD cohorts

the study is novel and of interest to the research community; strong dataset

Suggestions:

- as written its largely a methodological paper (thats fine); expand on how this would be important for clinicians - what are the practical consequences of this variability? How can this change or. influence referral or CKD classification?

*We agree that the clinical implications should be made clearer. We have expanded the Discussion (lines 351 to 366) emphasize that the variability quantified in this study is directly relevant to clinical interpretation of eGFR, especially near decision thresholds used for CKD staging, referral to nephrology, drug dosing, and confirmatory testing. Our findings do not suggest that clinicians should abandon pooled CKD-EPI equations. Rather, they support interpreting eGFR as an estimate with uncertainty, particularly when values are close to clinically meaningful cut points. We have added text noting that variability from study-level differences, patient-level non-GFR determinants of creatinine/cystatin C, and mGFR measurement error can contribute to reclassification around thresholds such as eGFR 60 or 30 ml/min/1.73m², and that this reinforces guideline recommendations to use confirmatory testing when clinical decisions are sensitive to small differences in eGFR.*

-did the investigators consider examining any other strata ? (diabetes vs non; CV disease vs non?)

*We thank the reviewer for raising this point. We did examine whether additional population-level factors explained study-level variation, although these results were not included in the manuscript to conserve space and maintain focus on the primary CKD versus non-CKD comparison. Specifically, we assessed race, geography, mGFR measurement method, BMI, and diabetes status. Cardiovascular disease status was not uniformly available across studies and therefore was not examined.*

*For each potential modifier, we fit two models. First, we added a main effect for the variable of interest to the creatinine-based random-effects model. Second, we allowed all population-level fixed-effect terms for creatinine, age, and sex to vary by that variable by including interaction terms. Separate models were fit for each potential modifier. We then compared the model with only the main effect to the model with interaction terms using a likelihood ratio test.*

*These analyses did not materially change our conclusions. Race, diabetes, and BMI had statistically significant main effects, but the estimated differences were modest (see* ***Table****), and there was no evidence that the creatinine, age, and sex coefficients varied meaningfully by any of the evaluated factors. For example, the average percent difference in GFR was 10.1% for Black versus non-Black participants, -1.7% for diabetes versus no diabetes, and 2.0% per 2 standard deviations of BMI. However, interaction p values were not significant for any factor examined, indicating the relationship of current terms did not differ by these factors. We therefore did not include these analyses in the manuscript.*

**Table.** We assessed the influence of additional population-level terms in two models: we first added main effects for race (Black vs. non-Black), geography (North America vs. Europe), mGFR measurement method (iohexol vs. iothalamate/Cr-EDTA), BMI, or diabetes status (yes vs. no), and second, we further allowed all population-level terms to vary by these groups. The average percent (%) difference in eGFR by group is given by the main effects. Separate models were performed for potential modifiers. All models included fixed effects for log serum creatinine with spline terms, age, sex, and deviations for studies unenriched for CKD.

| **Model** | **Average % Difference in GFR (95% CI)** | **P^1^** | **P int^2^** |
| --- | --- | --- | --- |
| Race (Black vs non-Black) | 10.1 (8.7, 11.6) | <.0001 | 0.28 |
| Geography (European vs. North America) | 2.7 (-5.4, 11.5) | 0.52 | 1 |
| mGFR method (iohexol vs. iothalamate/Cr-EDTA) | 2.6 (-2.5, 7.9) | 0.33 | 1 |
| Diabetes (yes vs. no) | -1.7 (-3.1, -0.3) | 0.02 | 1 |
| BMI (per 2 standard deviations in kg/m^2^) | 2.0 (1.2, 2.8) | <.0001 | 1 |

^1^P value for main effect.

^2^P value for interaction. Interaction p values were calculated by taking twice the difference in log-likelihood between the first model which included only main effects for the given variable and the second model which allowed all fixed effect coefficients to vary by the given variable and comparing to a $\chi^{2}$ distribution with 9 degrees of freedom.

-error variance is assumed to be 0.015 - any other thresholds examined?

*The value of 0.015 was selected a priori based on repeated GFR measurements in two studies, as described in the Supplement. We note in the discussion that error in mGFR has also been reported to be as low as 5%. If the true error in mGFR is in fact closer to 5%, a larger proportion of the total variation in mGFR would be attributed to within-study factors, while the proportion explained by between-study factors would remain unchanged. For example, for a 50-year-old female with a serum creatinine of 2.0 mg/dL, we estimated the total variance in mGFR to be 0.071, with 18.4% attributable to measurement error in mGFR, 54.1% to within-study variation, and 27.4% to between-study variation. If the CV for mGFR were instead 5%, only 3.5% of the total variance would be explained by measurement error, while 69.1% and 27.4% would be explained by within-study and between-study factors, respectively. Studies that report CVs closer to 5% may reflect shorter time intervals between repeat assessments than those included here. We have revised the Discussion to reflect these points (lines 352–360).*

-why do pooled equations work so well despite such large variation?

*This is an important point, and we have revised the Discussion (lines 317 – 326) to explain it more clearly. Although there was substantial study-level variation, much of this variation was balanced across the diverse studies in the CKD-EPI pooled database. The pooled equation estimates the average relationship across these populations, which is appropriate for broad clinical use when the clinical context of a new patient is uncertain. In addition, the CKD-EPI functional form appears to capture the dominant relationship between filtration markers, age, sex, and mGFR, while random-effects models mainly quantify additional uncertainty around that average relationship rather than producing a substantially different population-average equation. Finally, pooled equations reduce overfitting compared with single-study equations, which may fit their source population well but generalize poorly.*

-whats BLUP? a sentence to explain its purpose

*We added a brief explanation of BLUP in the Results(lines 207-210). BLUPs are the random-effects model estimates of each study’s study-specific coefficients, which combine information from that study with information from the full pooled dataset. They are useful here because they provide stabilized study-specific estimates while avoiding the instability of fitting each study entirely separately, especially for smaller studies.*

-should Figure 6 be the main figure? moved earlier?

*We agree that Figure 6 conveys one of the most important messages of the paper: equations developed within a single study can substantially underestimate external prediction error. We considered moving Figure 6 earlier. However, because Figures 1–5 build the rationale for the model, variance decomposition, and comparison with CKD-EPI equations, we have retained the figure order. If the editor prefers a shorter or more clinically oriented presentation, Figure 6 could also be moved earlier or emphasized as the key summary figure.*

Reviewer #2: Thank you for the opportunity to review this manuscript about the impact of study level heterogeneity on the development and performance of glomerular filtration rate estimating equations. The authors leverage a collection of CKD-EPI development and validation cohorts and apply appropriate hierarchical modelling techniques to quantify sources of variability.

The primary concern relates to the framing of the manuscript. The introduction and discussion suggest that the findings have important implications for future equation development. However, the principal conclusion is that the current pooled CKD-EPI approach remains appropriate despite measurable study level heterogeneity. The manuscript would be strengthened by tempering claims of novelty and emphasizing that the analyses primarily provide empirical validation of the existing pooled approach rather than identifying an alternative modelling strategy.

*We agree that the manuscript should be framed less as proposing an alternative strategy for future equation development and more as an empirical evaluation of study-level heterogeneity within the existing CKD-EPI pooled-data framework. We have revised the Introduction, Abstract, and Discussion to temper claims about implications for future equation development.*

The clinical implications should also be more clearly articulated. Although the authors demonstrate that study level variability exists, it is not clear how these findings should influence clinical interpretation of estimated GFR, future equation development, or research priorities. A stronger discussion of the practical implications would improve the overall impact of the manuscript.

*As indicated in our response to reviewer #1, we have expanded the clinical implications in the discussion.*

The classification of studies into CKD and non-CKD populations is relatively broad and likely masks important biological differences among study populations. Healthy kidney donor candidates, transplant recipients, patients with diabetes, and other disease specific cohorts may contribute differently to the observed heterogeneity. Additional justification for this classification would strengthen interpretation.

*We agree there is considerable heterogeneity within the non‑CKD populations and, over the course of preparation of this manuscript, we have revised our classification of these studies several times. It is important to note that the non‑CKD populations include studies that were not designed with any special consideration of CKD status; therefore, some patients within these studies do in fact have CKD. We added more details to the methods to clarify this (lines 127-129).*

*We consider using a three-level categorization: studies of populations with CKD, studies of populations at risk for CKD, and studies of healthy populations; however, we opted for the two-level categorization to simplify the model and our conclusions. We also explored whether adding factors such as race, mGFR measurement method, geography, and BMI could further explain the within-study variation (see Table above); however, these adjustments produced only small changes in the model error estimates. We therefore chose to omit these results to conserve space and maintain focus in the current version of the paper.*

*We note in the limitations that the categorization of CKD versus non-CKD is not perfect and does not fully capture the heterogeneity within each group, and residual heterogeneity may partially contribute to the observed study-level differences.*

The manuscript would also benefit from several clarifications. The rationale for the selected random effects structure should be described in greater detail, uncertainty around the estimated variance components should be acknowledged, and the ‘leave one study out’ analyses should be presented as internal validation rather than true external validation. Reporting performance measures such as bias, calibration, and P30 alongside RMSE would provide a more complete assessment of model performance and should be acknowledged as limitations of the report.

*We thank the reviewer for these helpful suggestions. We have revised the manuscript and supplement to clarify the rationale for the selected random-effects structure (lines 166-174). Specifically, we now state more explicitly that the model was designed to separate systematic differences between CKD and non-CKD study populations from additional unexplained study-level deviations, while avoiding an overly complex random-effects structure that would be unstable within smaller studies. We used random effects for study-specific intercepts and slopes for the main marker, age, and sex, but included only a single random slope for creatinine or cystatin C rather than separate random effects for each spline term, because many studies had limited marker distributions on one side of the knot.*

*We also agree that the estimated variance components are subject to uncertainty. We have revised the text to acknowledge that the variance decompositions should be interpreted as model-based estimates rather than exact partitions, and that uncertainty in these components is not fully reflected in the graphical summaries (lines 187-190).*

*We further agree with the reviewer that the leave-one-study-out analyses are not equivalent to validation in a fully independent external dataset. We have revised the manuscript to describe these analyses as* ***internal cross-study validation*** *rather than true external validation.*

*Finally, we agree that additional performance measures such as bias, calibration, and P30 would provide complementary information about model performance. Because the goal of this analysis was to quantify sources of variation and compare relative prediction error across modeling strategies, we decided to focus on RMSE as a summary measure of total error on the log scale.*

Minor points: The discussion is repetitive, several sentences are unnecessarily long, and there are minor grammatical errors throughout the text. Figure 5 is difficult to interpret because of the number of overlapping elements, whereas Figure 6 effectively communicates the principal findings.

*We apologize for the grammar errors and long sentences. We have revised the manuscript with this in mind.*
