# Supplementary material for: Study variation in estimating Glomerular Filtration Rate (GFR) from serum creatinine, cystatin C, age, and sex
Source: PLoS One. 2026 Sep 28;21(9):e0357209. doi: 10.1371/journal.pone.0357209 (PMC13618964; doi:10.1371/journal.pone.0357209)
Supplement: Peer review history

# Peer review: Decision Letter 0

**DOI:** 10.1371/journal.pone.0357209.r001

**Authors:** Ann Bugeja

5 Jul 2026

Study variation in estimating Glomerular Filtration Rate (GFR) from serum creatinine, cystatin C, age, and sex

PLOS One

Dear Dr. FINO,

Thank you for submitting your manuscript to PLOS ONE. After careful consideration, we feel that it has merit but does not fully meet PLOS ONE’s publication criteria as it currently stands. Therefore, we invite you to submit a revised version of the manuscript that addresses the points raised during the review process.

This manuscript presents a novel and methodologically rigorous analysis examining the sources of variation between measured and estimated GFR using a unique pooled CKD-EPI dataset. The study is of clear interest to the nephrology research community and demonstrates that study-level heterogeneity, measurement error, and patient-level variation all contribute to discrepancies between mGFR and eGFR, with differing patterns observed in CKD and non-CKD populations. The statistical methods are appropriate, and the overall conclusion that pooled equations remain preferable to single-study equations is well supported. However, the manuscript would benefit from placing greater emphasis on the clinical implications of these findings. Specifically, the authors should discuss how this variability might influence CKD classification, referral decisions, or clinical interpretation of eGFR, and explain why pooled equations continue to perform well despite substantial study-level heterogeneity. Additional justification for the broad CKD versus non-CKD classification, consideration of other clinically relevant subgroup analyses (such as diabetes or cardiovascular disease) would strengthen the manuscript. The methods section would also benefit from a brief explanation of best linear unbiased prediction (BLUP) for readers unfamiliar with mixed-effects modelling. Finally, the presentation could be improved by simplifying portions of the discussion, reducing repetition, and considering whether Figure 6, which most clearly conveys the principal findings, should be moved earlier in the manuscript or highlighted as a primary figure.

Please submit your revised manuscript by Aug 04 2026 11:59PM. If you will need more time than this to complete your revisions, please reply to this message or contact the journal office at plosone@plos.org. When you're ready to submit your revision, log on to https://www.editorialmanager.com/pone/ and select the 'Submissions Needing Revision' folder to locate your manuscript file.

Please include the following items when submitting your revised manuscript:

- A letter that responds to each point raised by the academic editor and reviewer(s). You should upload this letter as a separate file labeled 'Response to Reviewers'.
- A marked-up copy of your manuscript that highlights changes made to the original version. You should upload this as a separate file labeled 'Revised Manuscript with Track Changes'.
- An unmarked version of your revised paper without tracked changes. You should upload this as a separate file labeled 'Manuscript'.

If you would like to make changes to your financial disclosure, please include your updated statement in your cover letter. Guidelines for resubmitting your figure files are available below the reviewer comments at the end of this letter.

If applicable, we recommend that you deposit your laboratory protocols in protocols.io to enhance the reproducibility of your results. Protocols.io assigns your protocol its own identifier (DOI) so that it can be cited independently in the future. For instructions see: https://journals.plos.org/plosone/s/submission-guidelines#loc-laboratory-protocols. Additionally, PLOS ONE offers an option for publishing peer-reviewed Lab Protocol articles, which describe protocols hosted on protocols.io. Read more information on sharing protocols at https://plos.org/protocols?utm_medium=editorial-email&utm_source=authorletters&utm_campaign=protocols.

As the corresponding author, your ORCID iD is verified in the submission system and will appear in the published article. PLOS supports the use of ORCID, and we encourage all coauthors to register for an ORCID iD and use it as well. Please encourage your coauthors to verify their ORCID iD within the submission system before final acceptance, as unverified ORCID iDs will not appear in the published article. *Only*  the individual author can complete the verification step; PLOS staff *cannot*  verify ORCID iDs on behalf of authors.

We look forward to receiving your revised manuscript.

Kind regards,

Ann Bugeja

Academic Editor

PLOS One

Journal Requirements:

When submitting your revision, we need you to address these additional requirements.

1. Please ensure that your manuscript meets PLOS ONE's style requirements, including those for file naming. The PLOS ONE style templates can be found at

https://journals.plos.org/plosone/s/file?id=wjVg/PLOSOne_formatting_sample_main_body.pdf and

https://journals.plos.org/plosone/s/file?id=ba62/PLOSOne_formatting_sample_title_authors_affiliations.pdf

2. Thank you for stating the following in the Competing Interests section: [Dr. Inker reports research grants to institution from the National Institute of Health/National Institute of Diabetes and Digestive and Kidney Diseases (grant R01DK116790), National Kidney Foundation, Chinnocks, Alexion and Novartis; consulting fees from Diamtrix; royalties from UpToDate; and participation on the medical advisory council for Alport Foundation and the scientific advisory board for National Kidney Foundation.

Dr. Levey reports research grants to his institution from the National Institute of Diabetes and Digestive and Kidney Diseases and royalties from UpToDate.

Tom Greene receives research funding from the National Kidney Foundation, the Collaborative Study Group, the National Institutes of Health, and the Patient-Centered Outcomes Research Institute (PCORI).

Dr. Haaland reports consulting fees from the National Kidney Foundation].

Please confirm that this does not alter your adherence to all PLOS ONE policies on sharing data and materials, by including the following statement: ""This does not alter our adherence to  PLOS ONE policies on sharing data and materials.” (as detailed online in our guide for authors http://journals.plos.org/plosone/s/competing-interests).  If there are restrictions on sharing of data and/or materials, please state these. Please note that we cannot proceed with consideration of your article until this information has been declared.

Please include your updated Competing Interests statement in your cover letter; we will change the online submission form on your behalf.

3. For studies involving third-party data, we encourage authors to share any data specific to their analyses that they can legally distribute. PLOS recognizes, however, that authors may be using third-party data they do not have the rights to share. When third-party data cannot be publicly shared, authors must provide all information necessary for interested researchers to apply to gain access to the data. (https://journals.plos.org/plosone/s/data-availability#loc-acceptable-data-access-restrictions)

For any third-party data that the authors cannot legally distribute, they should include the following information in their Data Availability Statement upon submission:

1) A description of the data set and the third-party source

2) If applicable, verification of permission to use the data set

3) Confirmation of whether the authors received any special privileges in accessing the data that other researchers would not have

4) All necessary contact information others would need to apply to gain access to the data

4. Please include captions for your Supporting Information files at the end of your manuscript, and update any in-text citations to match accordingly. Please see our Supporting Information guidelines for more information: http://journals.plos.org/plosone/s/supporting-information.

5. If the reviewer comments include a recommendation to cite specific previously published works, please review and evaluate these publications to determine whether they are relevant and should be cited. There is no requirement to cite these works unless the editor has indicated otherwise.

6. Please review your reference list to ensure that it is complete and correct. If you have cited papers that have been retracted, please include the rationale for doing so in the manuscript text, or remove these references and replace them with relevant current references. Any changes to the reference list should be mentioned in the rebuttal letter that accompanies your revised manuscript. If you need to cite a retracted article, indicate the article’s retracted status in the References list and also include a citation and full reference for the retraction notice.

Additional Editor Comments:

This manuscript presents a novel and methodologically rigorous analysis examining the sources of variation between measured and estimated GFR using a unique pooled CKD-EPI dataset. The study is of clear interest to the nephrology research community and demonstrates that study-level heterogeneity, measurement error, and patient-level variation all contribute to discrepancies between mGFR and eGFR, with differing patterns observed in CKD and non-CKD populations. The statistical methods are appropriate, and the overall conclusion that pooled equations remain preferable to single-study equations is well supported. However, the manuscript would benefit from placing greater emphasis on the clinical implications of these findings. Specifically, the authors should discuss how this variability might influence CKD classification, referral decisions, or clinical interpretation of eGFR, and explain why pooled equations continue to perform well despite substantial study-level heterogeneity. Additional justification for the broad CKD versus non-CKD classification, consideration of other clinically relevant subgroup analyses (such as diabetes or cardiovascular disease) would strengthen the manuscript. The methods section would also benefit from a brief explanation of best linear unbiased prediction (BLUP) for readers unfamiliar with mixed-effects modelling. The presentation could also be improved by simplifying portions of the discussion, reducing repetition, and considering whether Figure 6, which most clearly conveys the principal findings, should be moved earlier in the manuscript or highlighted as a primary figure.

[Note: HTML markup is below. Please do not edit.]

Reviewers' comments:

Reviewer's Responses to Questions

**Comments to the Author**

1. Is the manuscript technically sound, and do the data support the conclusions?

Reviewer #1: Yes

Reviewer #2: Yes

**********

2. Has the statistical analysis been performed appropriately and rigorously?

Reviewer #1: Yes

Reviewer #2: Yes

**********

3. Have the authors made all data underlying the findings in their manuscript fully available?

The PLOS Data policy

Reviewer #1: Yes

Reviewer #2: Yes

**********

4. Is the manuscript presented in an intelligible fashion and written in standard English?

Reviewer #1: Yes

Reviewer #2: Yes

**********

Reviewer #1: interesting study that examines sources of variation between mGFR and eGFR; found differing sources of variation (study variation, measurement error, patient -level errors) in non CKD and CKD cohorts

the study is novel and of interest to the research community; strong dataset

Suggestions:

- as written its largely a methodological paper (thats fine); expand on how this would be important for clinicians - what are the practical consequences of this variability? How can this change or. influence referral or CKD classification?

-did the investigators consider examining any other strata ? (diabetes vs non; CV disease vs non?)

-error variance is assumed to be 0.015 - any other thresholds examined?

-why do pooled equations work so well despite such large variation?

-whats BLUP? a sentence to explain its purpose

-should Figure 6 be the main figure? moved earlier?

Reviewer #2: Thank you for the opportunity to review this manuscript about the impact of study level heterogeneity on the development and performance of glomerular filtration rate estimating equations. The authors leverage a collection of CKD-EPI development and validation cohorts and apply appropriate hierarchical modelling techniques to quantify sources of variability.

The primary concern relates to the framing of the manuscript. The introduction and discussion suggest that the findings have important implications for future equation development. However, the principal conclusion is that the current pooled CKD-EPI approach remains appropriate despite measurable study level heterogeneity. The manuscript would be strengthened by tempering claims of novelty and emphasizing that the analyses primarily provide empirical validation of the existing pooled approach rather than identifying an alternative modelling strategy.

The clinical implications should also be more clearly articulated. Although the authors demonstrate that study level variability exists, it is not clear how these findings should influence clinical interpretation of estimated GFR, future equation development, or research priorities. A stronger discussion of the practical implications would improve the overall impact of the manuscript.

The classification of studies into CKD and non-CKD populations is relatively broad and likely masks important biological differences among study populations. Healthy kidney donor candidates, transplant recipients, patients with diabetes, and other disease specific cohorts may contribute differently to the observed heterogeneity. Additional justification for this classification would strengthen interpretation.

The manuscript would also benefit from several clarifications. The rationale for the selected random effects structure should be described in greater detail, uncertainty around the estimated variance components should be acknowledged, and the ‘leave one study out’ analyses should be presented as internal validation rather than true external validation. Reporting performance measures such as bias, calibration, and P30 alongside RMSE would provide a more complete assessment of model performance and should be acknowledged as limitations of the report.

Minor points: The discussion is repetitive, several sentences are unnecessarily long, and there are minor grammatical errors throughout the text. Figure 5 is difficult to interpret because of the number of overlapping elements, whereas Figure 6 effectively communicates the principal findings.

**********

what does this mean?). If published, this will include your full peer review and any attached files.

If you choose “no”, your identity will remain anonymous but your review may still be made public.

**Do you want your identity to be public for this peer review?**  For information about this choice, including consent withdrawal, please see our Privacy Policy

Reviewer #1: **Yes:**  Manish M Sood

Reviewer #2: No

**********

[NOTE: If reviewer comments were submitted as an attachment file, they will be attached to this email and accessible via the submission site. Please log into your account, locate the manuscript record, and check for the action link "View Attachments". If this link does not appear, there are no attachment files.]

To ensure your figures meet our technical requirements, please review our figure guidelines: https://journals.plos.org/plosone/s/figures

You may also use PLOS’s free figure tool, NAAS, to help you prepare publication quality figures: https://journals.plos.org/plosone/s/figures#loc-tools-for-figure-preparation.

NAAS will assess whether your figures meet our technical requirements by comparing each figure against our figure specifications.

# Author comment: Author response to Decision Letter 1

**DOI:** 10.1371/journal.pone.0357209.r002

22 Jul 2026

Thank you very much for reviewing our paper. Please find our revised manuscript and responses to important points raised by the reviewers.

Attachment Submitted filename: Response to Reviewers.docx

# Peer review: Decision Letter 1

**DOI:** 10.1371/journal.pone.0357209.r003

**Authors:** Rajeevan Selvaratnam

13 Aug 2026

Study variation in estimating Glomerular Filtration Rate (GFR) from serum creatinine, cystatin C, age, and sex

PONE-D-26-26119R1

Dear Dr. FINO,

We’re pleased to inform you that your manuscript has been judged scientifically suitable for publication and will be formally accepted for publication once it meets all outstanding technical requirements.

Within one week, you’ll receive an e-mail detailing the required amendments. When these have been addressed, you’ll receive a formal acceptance letter and your manuscript will be scheduled for publication.

An invoice will be generated when your article is formally accepted. Please note, if your institution has a publishing partnership with PLOS and your article meets the relevant criteria, all or part of your publication costs will be covered. Please make sure your user information is up-to-date by logging into Editorial Manager at Editorial Manager® and clicking the ‘Update My Information' link at the top of the page. For questions related to billing, please contact billing support.

If your institution or institutions have a press office, please notify them about your upcoming paper to help maximize its impact. If they’ll be preparing press materials, please inform our press team as soon as possible -- no later than 48 hours after receiving the formal acceptance. Your manuscript will remain under strict press embargo until 2 pm Eastern Time on the date of publication. For more information, please contact onepress@plos.org.

Kind regards,

Rajeevan Selvaratnam

Academic Editor

PLOS One

Additional Editor Comments (optional):

Reviewers' comments:

Reviewer's Responses to Questions

**Comments to the Author**

Reviewer #1: All comments have been addressed

Reviewer #2: (No Response)

**********

2. Is the manuscript technically sound, and do the data support the conclusions?

Reviewer #1: Yes

Reviewer #2: Partly

**********

3. Has the statistical analysis been performed appropriately and rigorously?

Reviewer #1: Yes

Reviewer #2: N/A

**********

4. Have the authors made all data underlying the findings in their manuscript fully available?

The PLOS Data policy

Reviewer #1: Yes

Reviewer #2: No

**********

5. Is the manuscript presented in an intelligible fashion and written in standard English?

Reviewer #1: Yes

Reviewer #2: Yes

**********

Reviewer #1: The revisions were well done, and substantially improved the manuscript.

Reviewer #2: Please submit a point by point response letter and a red line document to highlight revisions and responses to reviewers.

**********

what does this mean?). If published, this will include your full peer review and any attached files.

If you choose “no”, your identity will remain anonymous but your review may still be made public.

**Do you want your identity to be public for this peer review?**  For information about this choice, including consent withdrawal, please see our Privacy Policy

Reviewer #1: **Yes:**  Manish M Sood

Reviewer #2: No

**********

# Editor report: Acceptance letter

**DOI:** 10.1371/journal.pone.0357209.r004

**Authors:** Rajeevan Selvaratnam

PONE-D-26-26119R1

PLOS One

Dear Dr. Fino,

I'm pleased to inform you that your manuscript has been deemed suitable for publication in PLOS One. Congratulations! Your manuscript is now being handed over to our production team.

At this stage, our production department will prepare your paper for publication. This includes ensuring the following:

* All references, tables, and figures are properly cited

* All relevant supporting information is included in the manuscript submission,

* There are no issues that prevent the paper from being properly typeset

You will receive further instructions from the production team, including instructions on how to review your proof when it is ready. Please keep in mind that we are working through a large volume of accepted articles, so please give us a few days to review your paper and let you know the next and final steps.

Lastly, if your institution or institutions have a press office, please let them know about your upcoming paper now to help maximize its impact. If they'll be preparing press materials, please inform our press team within the next 48 hours. Your manuscript will remain under strict press embargo until 2 pm Eastern Time on the date of publication. For more information, please contact onepress@plos.org.

You will receive an invoice from PLOS for your publication fee after your manuscript has reached the completed accept phase. If you receive an email requesting payment before acceptance or for any other service, this may be a phishing scheme. Learn how to identify phishing emails and protect your accounts at https://explore.plos.org/phishing.

If we can help with anything else, please email us at customercare@plos.org.

Thank you for submitting your work to PLOS One and supporting open access.

Kind regards,

PLOS One Editorial Office Staff

on behalf of

Dr. Rajeevan Selvaratnam

Academic Editor

PLOS One
